# Size-dependent activity of carbon dots for photocatalytic H_2_ generation in combination with a molecular Ni cocatalyst†

**DOI:** 10.1039/d3nr03300g

**Published:** 2023-08-28

**Authors:** Carla Casadevall, Ava Lage, Manting Mu, Heather F. Greer, Daniel Antón-García, Julea N. Butt, Lars J. C. Jeuken, Graeme W. Watson, Max García-Melchor, Erwin Reisner

**Affiliations:** a Yusuf Hamied Department of Chemistry, University of Cambridge Lensfield Road Cambridge CB2 1EW UK reisner@ch.cam.ac.uk; b School of Chemistry, Trinity College Dublin, College Green Dublin 2 Ireland; c School of Chemistry and School of Biological Sciences, University of East Anglia, Norwich Research Park Norwich NR4 7TJ UK; d Leiden Institute of Chemistry, Leiden University PO Box 9502 2300 RA Leiden The Netherlands; e CRANN and AMBER Research Centres, College Green Dublin 2 Ireland garciamm@tcd.ie

## Abstract

Carbon dots (CDs) are low-cost light-absorbers in photocatalytic multicomponent systems, but their wide size distribution has hampered rational design and the identification of the factors that lead to their best performance. To address this challenge, we report herein the use of gel filtration size exclusion chromatography to separate amorphous, graphitic, and graphitic N-doped CDs depending on their lateral size to study the effect of their size on photocatalytic H_2_ evolution with a DuBois-type Ni cocatalyst. Transmission electron microscopy and dynamic light scattering confirm the size-dependent separation of the CDs, whereas UV-vis and fluorescence spectroscopy of the more monodisperse fractions show a distinct response which computational modelling attributes to a complex interplay between CD size and optical properties. A size-dependent effect on the photocatalytic H_2_ evolution performance of the CDs in combination with a molecular Ni cocatalyst is demonstrated with a maximum activity at approximately 2–3 nm CD diameter. Overall, size separation leads to a two-fold increase in the specific photocatalytic activity for H_2_ evolution using the monodisperse CDs compared to the as synthesized polydisperse samples, highlighting the size-dependent effect on photocatalytic performance.

## Abstract 2

We demonstrate that purification and size separation of polydisperse carbon dot materials unveils the real active nanoparticles and enhances the photocatalytic H_2_ evolution using monodisperse carbon dots.

## Graphical abstract

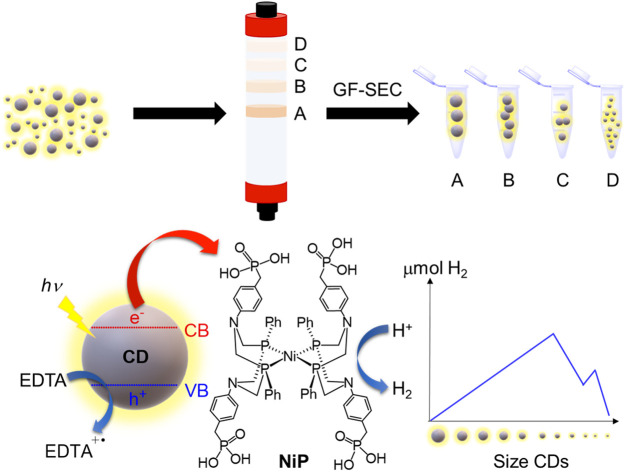

## 1. Introduction

Carbon dots (CDs) are a class of photoluminescent pseudo-spherical nanoparticles with sizes typically ranging from 1 to 10 nm. They generally consist of a carbonaceous core stabilized by oxidized surface groups such as carboxylic acids and alcohols.^1^ CDs have recently garnered increasing interest as they are easy to synthesize and functionalize, biocompatible, environmentally benign, robust, water soluble, while displaying excellent photoluminescence and fluorescence properties.^2–4^ These advantageous properties have prompted investigations of CDs for many different applications, ranging from biomedicine to (opto)electronics.^5–9^

The photocatalytic activity of CDs in combination with cocatalysts also renders these materials as promising light-absorbers for the light-driven synthesis of fuels and chemicals.^2,10–12^ However, the rational design and development of CDs has been obscured by their use as polydisperse materials, making the rigorous analysis and identification of the truly active material challenging. Furthermore, the rich variety of organic precursors and synthetic procedures available (*e.g.*, hydrothermal, microwave, pyrolysis) results in a broad range of different CD materials and heterogeneous fractions with wide size distributions that can influence their physicochemical properties. CD materials are also commonly accompanied by unreacted starting materials or by-products that can affect their photoluminescence and mask their real light absorption properties.^13–15^ Consequently, the overall optical properties are the result of different-size fractions, potentially obscuring the real photocatalytic species.^13–15^

A purification step can provide a more homogeneous size and allows for the characterization of pure and monodisperse CDs to understand the origin of their activity.^16^ Common purification techniques include solid phase extraction on alumina,^17^ reverse micelle methods,^18^ and silica column chromatography;^19–21^ or when size-separation is to be accomplished, dialysis,^13,22^ ultrafiltration,^23^ gel electrophoresis,^24,25^ high performance liquid chromatography (HPLC),^26^ size-selective precipitation,^27^ and more recently, size exclusion chromatography (SEC).^14,28,29^ However, the efficient large scale purification of CDs, even with well dispersed samples in water, remains a challenge.^14^ Furthermore, to the best of our knowledge, a systematic study of purified and monodisperse CDs in photocatalysis has not been reported to date.

**Scheme 1:**
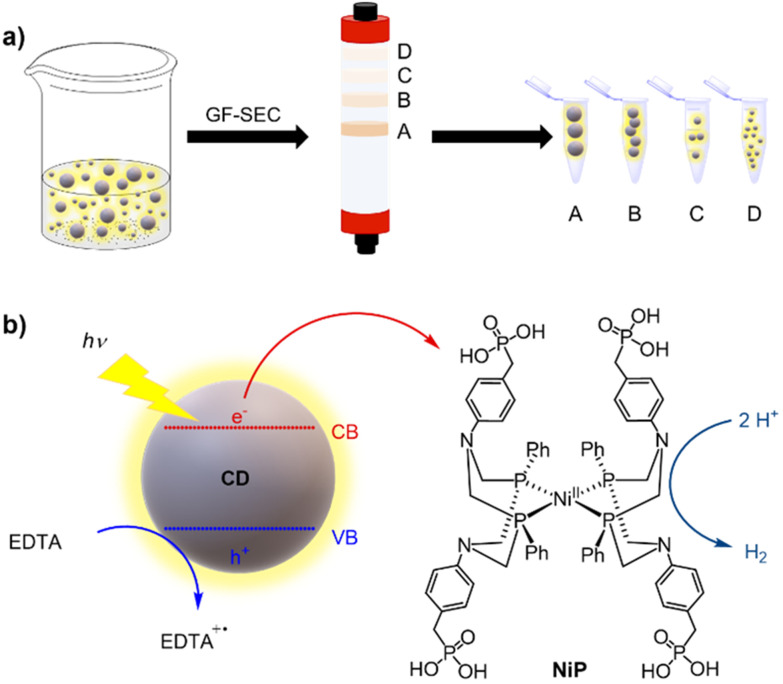
Illustration of the (a) GF-SEC purification of the synthesized CDs used in this work, and (b) HER activity with NiP as cocatalyst using EDTA as sacrificial electron donor. VB and CB refer to the valence and conduction bands, respectively.

In this work, we applied gel filtration size exclusion chromatography (GF-SEC) to purify CDs and separate them by their size to study their intrinsic photocatalytic activity (Scheme 1a). The choice of GF-SEC was inspired by previous reports showing the efficient purification of hydrocarbons and polymers even on a large scale.^30–32^ Hence, we selected three types of CDs which had previously been shown to promote photocatalytic H_2_ production when combined with H_2_ evolving catalysts, namely amorphous (a-CD),^33^ graphitic (g-CD), and graphitic nitrogen-doped (g-N-CD) CDs.^10^ After purifying the CDs by GF-SEC, we characterized them by UV-vis and fluorescence spectroscopy, as well as dynamic light scattering (DLS), zeta potential, fourier transform infrared spectroscopy (FTIR), and transmission electron microscopy (TEM). These measurements were complemented by computational time-dependent density functional theory (TD-DFT) studies on model graphene clusters with different sizes and thicknesses aimed at rationalizing the trends observed in the recorded UV-vis spectra of the size separated CDs. Finally, the size-dependent photocatalytic activity towards the hydrogen evolution reaction (HER) was studied using the well-known DuBois-type molecular Ni cocatalyst containing a [Ni(P_2_^*R*′^N_2_^*R*′′^)_2_]^2+^ core (P_2_^*R*′^N_2_^*R'*′^ = bis(1,5-*R*′-diphospha-3,7-*R*′′-diazacyclooctane)) with water-solubilizing phosphonic acid groups (NiP)^34,35^ in aqueous solution with ethylenediaminetetraacetic acid (EDTA) as a sacrificial electron donor (SED) (Scheme 1b).

## 2. Experimental section

### 2.1. Materials

The quality of the employed gases for synthesis (N_2_ and Ar) was 99.9995%, while the nitrogen gas used in the purging stations had 2% methane content (BOC). The following reagents and solvents were purchased from commercial sources and used as received, unless otherwise stated: deuterated solvents (Sigma Aldrich, 99.9 atom % D), ethanol (VWR Chemicals), hydrochloric acid (Sigma Aldrich, >95%), boric acid (Sigma Aldrich), ethylenediaminetetraacetic acid (EDTA, Sigma Aldrich), sodium chloride (Fisher Chemical), sodium hydroxide (Fisher Chemical), tris(hydroxymethyl)aminomethane (Tris, Sigma Aldrich). Millipore water (18.2 MΩ cm at 25 °C) was used throughout this work. Buffer solutions were made using analytical grade reagents and titrated to the desired pH, as determined by a pH electrode (Mettler Toledo; SevenEasy) using NaOH or HCl, as needed. Solvents used for synthesis, such as tetrahydrofuran (THF), and dichloromethane (DCM), were distilled on sodium (THF) or calcium hydride (DCM) before use. The NiP cocatalyst was synthesized following a reported procedure.^34,35^

### 2.2. Physical methods

UV–visible spectroscopy was carried out on a Cary 60 UV–vis spectrometer using quartz cuvettes with 1 cm path length from Thorlabs. Infrared spectra were obtained with a Thermo Scientific Nicolet iS50 FTIR spectrometer. Emission spectra were recorded using a spectrofluorometer (FS5 Spectrofluorometer, Edinburgh Instrument). A gas-tight quartz cuvette with a path length of 1 cm was used (from Thorlabs) under N_2_ atmosphere at 298 K and CD materials were excited at 360 and 405 nm. TEM images were collected using a Thermo Scientific (FEI) Talos F200X G2 TEM operating at 200 kV. TEM images were acquired using a Ceta 16M CMOS camera. Samples were prepared by applying 5 μL of the suspended sample in aqueous solution onto continuous carbon 300 mesh Cu grids that were negative glow discharged using a Quorum Technologies GloQube. DLS and zeta potential studies were performed in a Zetasizer Nano ZS spectrometer using a red laser (632.8 nm). The produced hydrogen in the photocatalytic reactions was analysed by gas chromatography (GC). Gases in the headspace were analysed by using a Shimadzu Tracera GC2010 Plus gas chromatograph using a barrier ionization discharge (BID) detector and a molsieve column (kept at 130 °C) using He as the carrier gas to quantify the amount of H_2_ produced. Aliquots of the gas in the headspace (50 μL) were taken at regular intervals during the photocatalytic experiments.

### 2.3. Synthesis and purification of CDs

CDs were synthesized and characterized following previously reported procedures.^10,33^ Shortly, citric acid was pyrolyzed in air at 180 °C for 40 h to obtain a-CD, and further heated to 320 °C for 100 h to yield g-CD. Finally, g-N-CD was synthesized by pyrolysis of aspartic acid in air at 320 °C for 100 h. Dissolution in water (50 mg mL^−1^ for all studied CDs) and NaOH solution (25 mM, for g-CD and g-N-CD only) produced a dark brown solution, which was passed through a microfilter (0.22 μm) and freeze-dried to obtain a brown solid. The materials were characterized by FTIR, UV-Vis and fluorescence spectroscopies and TEM.

Then, an Äkta purifier (ÄKTA pure 150, GE Healthcare) was used for CD purification. For the large-scale purification of the CDs, we used a Superdex 200 pg HiLoad 26/600 GL prepacked SEC column (Cytiva). Superdex resins have a range of pore sizes, and the range of protein sizes that can be separated is typically specified in terms of the molecular weight. For this column, the resolution of the resin was specified as 10–600 kDa, which for a protein average density of 1.37 g cm^−3^ equates to 2.8–11 nm (diameter). As such, the pore size of the resin can be assumed to be mostly around these sizes, which corresponds to the CDs diameters studied in this work. Unless otherwise specified, the column was equilibrated overnight with 20 mM borate buffer (pH 8, prepared from boric acid and sodium borate) (2 column volumes – 640 mL). A buffered solution of the bulk CDs (a-CDs, g-CDs or g-N-CDs, up to 13 mL) was then injected into the column *via* a loop. The column was eluted at a flow rate of 2 mL min^−1^; the eluate was monitored at 220, 280 and 530 nm and collected in aliquots of 4 mL each. After elution of the sample, the column was washed with (2 volumes) borate buffer. Fractions were analysed by TEM as well as UV-Vis and fluorescence spectroscopies, and DLS in borate buffer pH 8 (20 mM), unless otherwise indicated.

### 2.4. General procedure for the photocatalytic studies with NiP

All photocatalytic reactions were conducted in a 20 mL septum-capped vial under vigorous stirring using an orbital stirrer and irradiating at 405 nm for 48 h under N_2_ containing 2% CH_4_ as a GC standard (purged for 15 min prior to irradiation). Catalytic photoreductions were performed in H_2_O (5 mL), CD (0.5 mg), NiP (10 μM, 50 nmol) and 0.1 M EDTA at pH 6. After sealing the reaction vessel, it was purged with nitrogen containing 2% methane as internal gas standard for further analysis and the produced gases were quantified in the reaction headspace. A LED photoreactor (*λ* = 405 ± 10 nm, 2.2 W intensity per reaction)^36^ was employed as light source. At indicated times, an aliquot of the reaction headspace was subjected to GC-BID analysis (see above). Reported yields are an average of at least three runs.

### 2.5. Computational details

#### Computational methods

Density functional theory (DFT) calculations reported in this work were carried out using the Gaussian09 software^37^ and the dispersion-corrected hybrid exchange–correlation functional ωB97X-D.^38^ Carbon and hydrogen atoms were described with the Pople's double-zeta 6-31G(d,p) basis set with polarization functions. Geometry optimizations were performed in solution (water, *ε* = 78.3553) using the implicit SMD solvation model^39^ and without imposing any symmetry constraints. Forces and atomic displacements were minimized with the ‘Tight’ optimization convergence criteria and the ‘Ultrafine’ grid for numerical integrations. The optimized structures were verified to be true minima on the potential energy surface by means of vibrational frequency analysis, confirming the existence of only real vibrational modes.

TD-DFT calculations were performed using the same settings as the geometry optimizations. Between 50–100 singlet states were calculated for each system depending on the cluster size, with the adsorption spectra calculated from the resulting excitation energies and oscillator strengths.

#### Graphene cluster packings

The stacking of the graphene sheets was initially investigated with the 2 × 2 system by modelling two layers with AA and AB packings (Fig. S15, ESI†). The geometry optimization of the former resulted in the structure with the AB stacking, and therefore, this packing was assumed for all systems containing two layers. Next, the ABA and ABC packings were investigated using the 3 × 3 system with three atomic layers. In this case, the Gibbs energy difference between these structures was 1.1 kcal mol^−1^ in favour of the ABA stacking. This small energy difference is in line with the coexistence of hexagonal and rhombohedral graphite structures with ABA and ABC packings, respectively, reported in the literature.^40^ Hence, the ABA packing was assumed for all the systems containing three or more atomic layers.

#### Excitation energies, character, oscillator strengths and optical adsorption

Excitations for all the systems were examined. TD-DFT vertical excitations at lower energies generally involved molecular orbitals from HOMO−3 to LUMO+3. Vertical excitations were much lower than HOMO–LUMO gaps. Tables S3 and S4† show examples of the excitations with finite oscillator strengths for the 4 × 4 − 1 L and 4 × 4 − 4 L systems, with their optical absorption shown in Fig. S16 and S17,† and the relevant molecular orbitals shown in Fig. S18 and S19.† Orbital absorption spectra were calculated using a python script assuming a Gaussian broadening of 0.4 eV.

#### Computational data available

The cartesian coordinates and energies of all the modelled structures are available at the following ioChem-BD dataset: http://dx.doi.org/10.19061/iochem-bd-6-141.

## 3. Results and discussion

### 3.1. Synthesis and characterization of bulk CDs

The CDs were synthesized and characterized as previously reported (Scheme S1†).^10,33^ Citric acid was pyrolyzed in air at 180 °C for 40 h to obtain a-CD, and further heated to 320 °C for 100 h to yield g-CD. Then, g-N-CD was synthesized by pyrolysis of aspartic acid in air at 320 °C for 100 h. Dissolution in water (50 mg mL^−1^ for all CDs) and NaOH solution (25 mM, for g-CD and g-N-CD only) produced a dark brown solution, which was passed through a microfilter (0.22 μm) and freeze-dried to obtain a brown solid. FTIR, UV-Vis and fluorescence spectroscopies together with TEM characterization of the materials was consistent with previous reports (Fig. S1–S3, ESI†).^10,33^

### 3.2. GF-SEC and spectroscopic characterization

The as-synthesized a-CD, g-CD and g-N-CD were purified using a dextran-based GF-SEC column^41^ (Scheme 1a) in an automatic protein column chromatograph (ÄKTA pure protein column chromatograph). After optimizing the separation protocol with a small scale Superdex 200 pg Increase 10/300 GL SEC column, a Superdex 200 pg HiLoad 26/600 GL (pore size 2.8–11 nm) column was used to separate the CDs on a gram scale. A borate buffered aqueous solution (20 mM, pH 8) as the eluent minimized the interactions between the column matrix and CDs, especially for g-CD and g-N-CD (Fig. S4, ESI†).^30^ The later elution times for a-CD as compared to g-CD and g-N-CD can therefore be explained by the stronger interactions between the a-CDs and the dextran gel matrix compared to the other CDs, despite using borate buffer. These CD-gel interactions do not change for the same type of CDs with different sizes, and hence, we can still employ GF-SEC for size-separation. After GF-SEC, all the fractions were freeze-dried to recover the size-separated CD materials and characterized by UV-vis and fluorescence spectroscopies, TEM, DLS, zeta potential and FTIR.

**Fig. 1:**
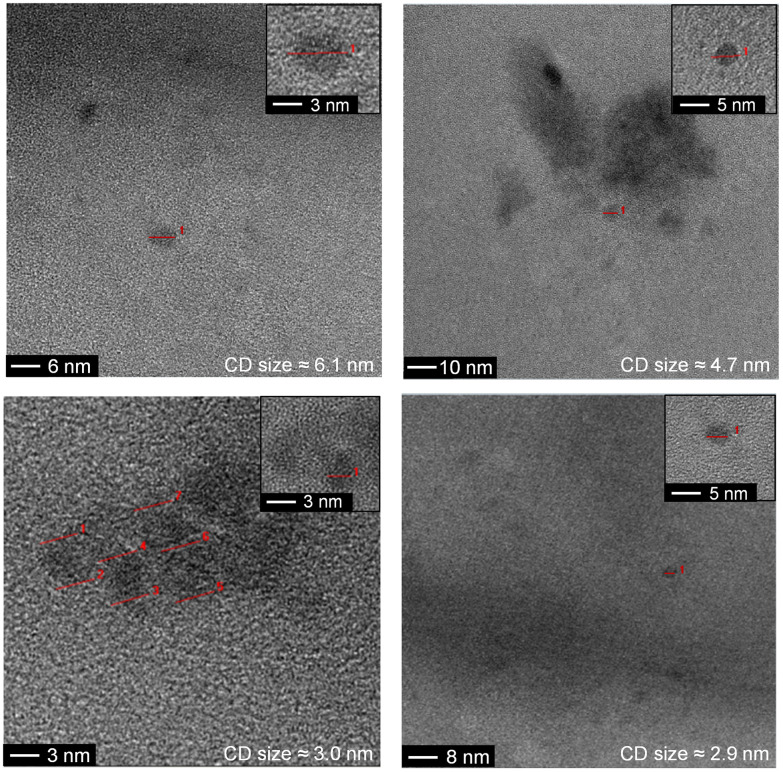
TEM images of the g-CD GF SEC separated fractions. Red lines indicate individual particles.

TEM characterization indicated different CD sizes in the different fractions (ranging from 7.6 to 2 nm); the larger elution volumes (longer elution times in the GF trace) contained CDs of smaller size, as expected (Fig. 1 and S6, S7b, S8, S9b and S10 in the ESI†). The low electron density of carbon particles and the appearance of aggregates in some TEM images, which are hypothesized to form during grid preparation, make accurate size determination difficult for some fractions (Fig. S7b, S8 and Table S1, ESI†). In the case of the g-CD samples, the CD size in the eluted fractions decreased from 6 to 1 nm (Fig. 1, S6 and Table S1, ESI†). A similar trend was observed for g-N-CD, with a decrease of the CD size in the eluted fractions from 4 to 1 nm (Fig. S9b–S10 and Table S1, ESI†), confirming a decrease in the CDs’ lateral size along the GF trace. DLS showed a trend of particle size decrease along the GF-SEC column for all the CD materials. However, the particles’ sizes were larger than observed by TEM, which suggests aggregate formation due to buffer interactions during DLS sample measurements^42^ (Table S2, ESI†).

**Fig. 2:**
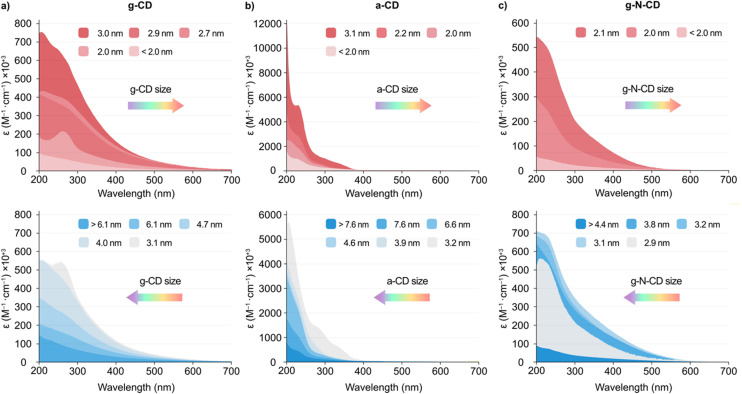
UV-vis spectra of the GF SEC separated fractions of (a) g-CD (size ranging from <2.0 to 3.0 nm -top- and from 3.1 to >6.1 nm -bottom- as described in the legend), (b) a-CD (size ranging from <2.0 to 3.1 nm -top- and from 3.2 to >7.6 nm -bottom- as described in the legend) and (c) g-N-CD (size ranging from <2.0 to 2.1 nm -top- and from 2.9 to >4.4 nm -bottom- as described in the legend) in borate buffer pH 8 (20 mM) at 25 °C. The direction of the arrows indicates the blue/red shift of the main bands observed with the different synthesized CDs.

The UV-vis and emission spectra for the different fractions revealed that the particle size influences the spectroscopic properties of the CDs. As a general trend, smaller particles displayed an increased light absorption per mass than larger CDs, with *ca.* 3 nm being the optimal size (Fig. 2 and S5, S7 and S9 in the ESI†). All three CD types displayed a non-monotonic behaviour for the absorption and photoluminescence (PL) peaks with particle size (Fig. 2 and S5, S7, S9, S11 and S12, ESI†). More specifically, a blueshift was observed in the absorption spectra with decreasing CDs size from 3.0 nm for g-CD, 3.1 nm for a-CD, and 2.1 nm for g-N-CD, respectively, down to *ca.* <2 nm. However, when the CDs size decreased from 6.1 to 3.1 nm for g-CD, 7.6 to 3.2 nm for a-CD, and 4.4 to 2.9 nm for g-N-CD, a redshift was observed (Fig. 2).

In addition, the emission spectra (*λ*_ex_ = 405 and 360 nm) of the size-separated a-CDs showed a slight redshift of the emission profile with a decrease in size from 3.1 to <2.0 nm, whereas a blueshift was observed with a decrease in size from 7.6 to 3.2 nm (Fig. S7d and S12a, ESI†). In contrast, the emission spectra for the g-CD and g-N-CD samples showed a blueshift upon decreasing the particle size with an increase in intensity from 2.7 to <2.0 nm and from 2.9 to <2.0 nm, respectively, whereas a redshift was observed with the decrease in size from >6.1 to 2.9 nm and from >4.4 to 3.1 nm for g-CD and g-N-CD, respectively (Fig. 2b and S9d, S12b–c, ESI†). This observation may suggest a blueshifted absorption edge in the graphitic CD series upon decreasing particle size in the nanometre scale, as reported for graphene quantum dots.^22^ Indeed, the energy of the band gap is inversely proportional to the size (*E*(eV) = 1/*L*, where *L* is the average lateral size of the CDs).^43^ In the case of g-CD and g-N-CD, for particle sizes below 3 nm, both the absorption and PL energy of the main bands decreased to 2 nm and then increased again, while for a-CD the PL energy increased again after 3 nm, after which it was maintained (Fig. S11–S13, ESI†). Therefore, the quantum confinement effect is not observed in this regime, as previously reported for graphene quantum dots.^22,43^ Additionally, in some of the excitation and emission spectra of the size separated CDs, we identified two bands irrespective of the excitation wavelength, presumably resulting from differently shaped nanoparticles, as previously documented (Fig. 2 and S12, ESI†).^22,43^

Finally, zeta potential measurements revealed an increase of the negative charge density in the particles with decreasing size when compared to the bulk materials (−42.5 *vs.* −17.0 mV, −52.7 *vs.* −26.8 mV and −40.2 *vs.* −23.0 mV for the smallest particles *vs.* bulk material for a-CD, g-CD and g-NCD, respectively (see Table S2, ESI†), suggesting the presence of more oxidized, negatively charged carboxylic acid groups per carbonaceous core in the smaller particles compared to the bulk materials.^10,33^ This was further supported by FTIR studies on the size-separated fractions for each type of CD. The different spectra show an increase in the intensity of the carbonyl stretch band with decreasing particle size (*ca.* 1700 and 1560 cm^−1^, 1560 and 1371 cm^−1^, and 1572 and 1367 cm^−1^ for a-CD, g-CD and g-N-CD, respectively), indicating a higher concentration of surface functional groups (negatively charged carboxylate groups mostly), and therefore an increased surface negative charge (Fig. S14, ESI†). This difference in charge density could also explain the non-monotonic optical properties due to differences in particle size and thickness.

### 3.3. Computational studies on model CD particles

**Fig. 3:**
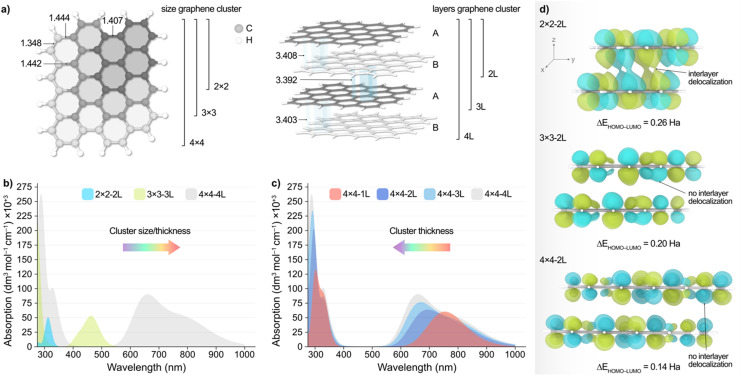
(a) Top (left) and side (right) view representations of the graphene clusters with different size and thickness employed in the calculations. Bond lengths and interlayer distances are given in Å. (b) Simulated absorption spectra of graphene particles with varying size and thickness. (c) Simulated absorption spectra of 4 × 4 graphene clusters with varying thickness. The direction of the arrows indicates the blue/red shift of the main bands observed with the different synthesized CDs. (d) Side view representation of the isosurfaces (isovalue = 0.02 a.u.) of the LUMO of the 2 × 2 (top), 3 × 3 (middle), and 4 × 4 (bottom) systems with two atomic layers. The calculated HOMO–LUMO gap (Δ*E*_HOMO–LUMO_) for each structure is also provided. Energy values are given in Hartrees (Ha).

To shed light into the non-monotonic behaviour observed in the UV-vis spectra of the separated CD fractions, we performed TD-DFT calculations at the ωB97X-D/6-31G(d,p) level of theory on different model clusters, as outlined in the computational details section. These structures, depicted in Fig. 3a, consisted of graphene sheets of various sizes and number of layers with an ABAB packing, ranging from 26 to 264 atoms. Other possible stackings (*i.e.* ABC and AAA) were also considered but were discarded based on their higher energies compared to the ABAB packing as aforementioned.

Fig. 3b, shows the simulated absorption spectra resulting from increasing both the size and thickness of the system, with 2 × 2 − 2 L being a 2 × 2 graphene cluster comprised of 2 × 2 hexagons of C atoms and 2 atomic layers in an AB packing. According to these results, decreasing the particle size/thickness leads to an overall blueshift in light absorption, in agreement with experiments. This observation, however, is made up of two contributions, namely the change in graphene sheet size and the number of atomic layers. Hence, to decouple these two effects, we modelled the absorption spectra of graphene particles wherein we independently varied their size and thickness, which is very challenging to accomplish experimentally.

Upon decreasing the graphene sheet size while maintaining the particle thickness to two atomic layers (Fig. S20, ESI†), we observed again a blueshift of the main low energy absorption peaks from *ca.* 250 nm. Conversely, Fig. 3c illustrates the effect of varying the thickness of the 4 × 4 graphene system, revealing a redshift of the energy absorption peaks as the number of layers decreases. Although less evident, this behaviour is also observed with the 3 × 3 model (Fig. S21b, ESI†), and can be rationalized with the conjugation of the π-system along the *xy* and *z* planes and the predicted HOMO–LUMO gap (Δ*E*_HOMO–LUMO_) upon varying the particle size and thickness, as we discuss in the following. We acknowledge that, while vertical excitations may involve a mix of molecular orbitals, we chose to use Δ*E*_HOMO–LUMO_ as a proxy since it can be directly obtained from the geometry relaxation. Fig. 3d shows the isosurfaces of the LUMO of the 2 × 2, 3 × 3, and 4 × 4 particles with two atomic layers, wherein it can be observed that the conjugation of the π-system increases along the *xy* plane as the graphene sheets become larger. This results in the red(blue)shift of the main absorption peaks with increasing (decreasing) sheet size, as observed experimentally. This is also supported by the calculated Δ*E*_HOMO–LUMO_ values (Fig. 3d), which decrease with the conjugation of the π-system, as expected. Conversely, we observed that increasing the number of layers has a different effect depending on the graphene system. Particularly, the absorption peaks in the 4 × 4 (Fig. 3c) and 3 × 3 structures blueshift with the increasing number of layers, while with the 2 × 2 model we see a blueshift for the absorption peaks until *ca.* 250 nm and a redshift beyond this wavelength (Fig. S21a, ESI†). We attribute this different behaviour with the smaller 2 × 2 − 2 L particle to the interplay between the lack of conjugation along the *xy* plane and the increased conjugation in the *z* direction (Fig. 3d and S22, ESI†), which is absent in the larger systems. In fact, increasing the particle thickness in the 3 × 3 and 4 × 4 structures results in a reduced intra- and interlayer conjugation (Fig. S23 and S24, ESI†), leading to the blueshift of the absorption peaks.

Altogether, TD-DFT calculations reveal a complex relationship between particle size/thickness and optical absorption, which may explain the non-monotonic dependence of both the absorption and PL energy peaks with the particle size of the eluted CD fractions. This unexpected trend might become particularly important in small CD particles where agglomeration is likely to occur. All these effects may also reflect in the catalytic applications of the CDs, as we discuss below.

### 3.4. Effect of CD size on photocatalytic activity

**Fig. 4:**
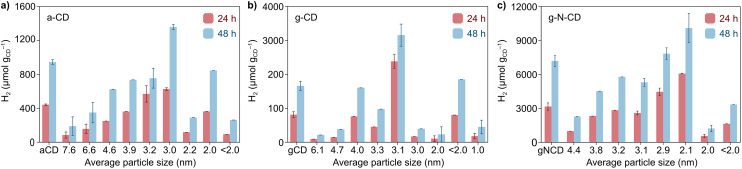
Comparison between the HER activity after 24 (red bars) and 48 h (blue bars) of the different size-separated fractions of the purified (a) a-CD, (b) g-CD, and (c) g-N-CD by GF-SEC in borate buffer pH 8 (20 mM) using a Superdex 200 pg HiLoad 26/600 GL column. Catalytic conditions: a-CD, g-CD and g-N-CD (0.5 mg), NiP (50 nmol), EDTA (0.1 M, pH 6) in water, irradiation at *λ* = 405 ± 10 nm, at 25 °C under N_2_ atmosphere.

After the detailed characterization of the size-separated fractions and the insights obtained from the TD-DFT simulations, we next explored whether their different size and spectroscopic properties can influence catalysis by examining their photochemical HER activity as a model reaction. Previous studies have reported CDs as efficient photosensitizers for HER in combination with NiP as a cocatalyst in aqueous solutions with EDTA as a SED.^10,33^ We performed light-driven HER studies with the size-separated a-CD, g-CD and g-N-CD (0.5 mg) samples using NiP as cocatalyst (50 nmol), EDTA (0.1 M, pH 6) in 5 mL total reaction volume, and irradiation using a thermostated LED photoreactor (*λ* = 405 ± 10 nm, 2.2 W intensity per reaction) at 25 °C.^36,44,45^ The photocatalytic studies, summarized in Fig. 4, showed that the CD size indeed affects the catalytic performance for H_2_ production. Although the size separation in the fractions is not 100% monodispersed, we do observe clear trends in HER activity, which are consistent with the absorption and emission spectra.

**Fig. 5:**
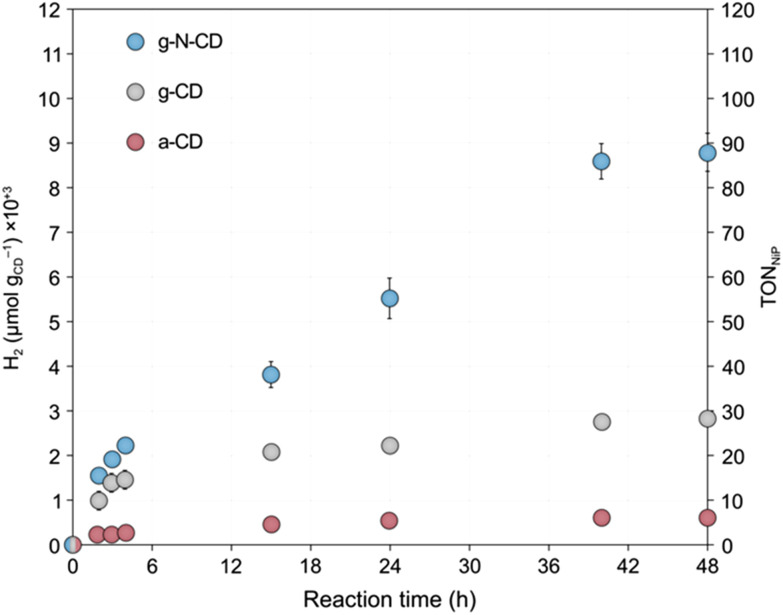
H_2_ evolution time traces with the optimum size for each type of CD prepared in this work after GF-SEC (3, 3.1 and 2.1 nm particle size for a-CD, g-CD and g-N-CD, respectively), using NiP as cocatalyst. Catalytic conditions: a-CD, g-CD and g-NCD (0.5 mg), NiP (50 nmol), and EDTA (0.1 M, pH 6) in water (5 mL reaction volume), irradiation at *λ* = 405 ± 10 nm, at 25 °C under N_2_ atmosphere for 48 h.

In particular, we found that HER activity increased with decreasing particle size of all types of CDs, until reaching a maximum and then decreased again with further reduction of the particle size. For a-CD and g-CD, the highest HER activity was obtained with the ca. 3 nm particles (Fig. 4a and b), which is consistent with the particle size with the highest absorption and PL peak energy (Fig. 2 and S11–S13, ESI†). Conversely, for g-N-CD, 2.9 and 2.1 nm particles displayed the highest HER activity (Fig. 4c), which also showed higher light excitation and emission. In all cases, the HER activity with g-N-CD was superior, as previously reported,^10^ and was maintained for more than 24 h levelling off after *ca.* 40 h (Fig. 4 and 5). Therefore, we attribute the enhanced HER activity observed with smaller CD particles to their higher light absorbance, presumably due to higher interlayer conjugation, as indicated by TD-DFT calculations.

## 4. Conclusions

We have demonstrated that GF-SEC is a reasonably effective purification technique to separate a-CD, g-CD and g-N-CD by their lateral size on a gram scale. GF-SEC has allowed us to deconvolute the different particle sizes composing the bulk material and to investigate each type of CD over a much narrower, well-defined size range. Both TEM and DLS support an overall decrease in the particle size along the GF traces, whereas UV-Vis and fluorescence spectroscopies showed that particle size influences the optical properties of the CDs.

Spectroscopic studies revealed a non-monotonic trend with decreasing particle size. Decreasing particle size from the largest CDs to those with a size of approximately 3 nm resulted in an increase in the band gap energy, consistent with a quantum confinement effect. However, this trend is reversed when particle size is further reduced, presumably due to the change in shape and thickness of particles at these smaller sizes, which prevents the description of this behaviour with band structures. In this context, TD-DFT calculations revealed an intricate relationship between particle size/thickness and optical absorption that correlates with the intra- and interlayer conjugation of the π-system, explaining the non-monotonic dependence of both the absorption and PL energy peaks with the particle size of the eluted fractions. Finally, light driven HER studies using NiP as cocatalyst showed a size-dependent effect. More specifically, we found that HER activity increases as the particle size decreases until reaching a maximum with an optimal particle size of *ca.* 3 nm for a-CD and g-CD and *ca.* 2 nm for g-N-CD. These trends can be rationalized by the higher light absorbance of the smaller CDs due to higher interlayer conjugation.

Overall, this work highlights the importance of purifying CDs to deconvolute the properties and activities of different sizes that compose the bulk CDs. The CDs' spectroscopic and light-harvesting properties allowed identifying the particle size that contributes the most to the catalytic activity. The approach described herein minimizes the risk of observing properties that arise from subproducts or other reaction impurities during the CDs synthesis. Furthermore, our purification protocol is general and straightforward and can be applied to study the properties of different types of CDs. The findings of our study provide compelling evidence that the polydisperse nature of CDs obscures the actual activity of individual CD size-separated fractions. This work serves as a motivation for researchers to prioritize size separation of CDs in future investigations to unlock their full potential and achieve superior performance in photocatalytic reactions.

## Author contributions

E. R. and C. C. directed and conceived this project with input from J. N. B. and L. J. C. J., C. C. synthesized the materials, performed most of the experimental work, and wrote the draft of the manuscript. C. C. and A. L. executed the DLS and zeta potential measurements. C. C. and H. F. G. performed the TEM measurements. D. A.-G. synthesized the NiP catalyst. M. G.-M. directed and performed the computational studies together with M. M. and G. W. W. All the authors discussed the results and contributed to writing and revising the manuscript.

## Conflicts of interest

The authors declare no conflict of interest.

## Supplementary Material

NR-015-D3NR03300G-s001
