# Supplementary material for: Size-dependent activity of carbon dots for photocatalytic H2 generation in combination with a molecular Ni cocatalyst
Source: Nanoscale. 2023 Aug 28;15(38):15775–84. doi: 10.1039/d3nr03300g (PMC10551879; doi:10.1039/d3nr03300g)
Supplement: NR-015-D3NR03300G-s001 [file NR-015-D3NR03300G-s001.pdf]

Electronic Supplementary Material (ESI) for Nanoscale.
This journal is © The Royal Society of Chemistry 2023

Electronic Supplementary Information

Size-dependent activity of carbon dots for photocatalytic H_2 generation in combination with a molecular Ni cocatalyst

Carla Casadevall,[a] Ava Lage,[a] Manting Mu,[b] Heather F. Greer,[a] Daniel Antón-García, [a] Julea N. Butt,[c] Lars J.C. Jeuken,[d] Graeme W. Watson,[b] Max García-Melchor,[b,e*] Erwin Reisner[a*]

a. Yusuf Hamied Department of Chemistry, University of Cambridge, Lensfield Road, Cambridge, CB2 1EW, UK.
b. School of Chemistry, Trinity College Dublin, College Green, Dublin 2, Ireland.
c. School of Chemistry and School of Biological Sciences, University of East Anglia, Norwich Research Park, Norwich, NR4 7TJ, UK.
d. Leiden Institute of Chemistry, Leiden University, PO Box 9502,2300 RA, Leiden, The Netherlands.
e. CRANN and AMBER Research Centres, College Green, Dublin 2, Ireland.
† Corresponding authors: reisner@ch.cam.ac.uk and garciamm@tcd.ie

Table of contents

1. Synthesis and characterization of CDs 2
2. Tables 3
3. Figures 6
4. References 19
End of Supporting Information 19

1. Synthesis and characterization of CDs

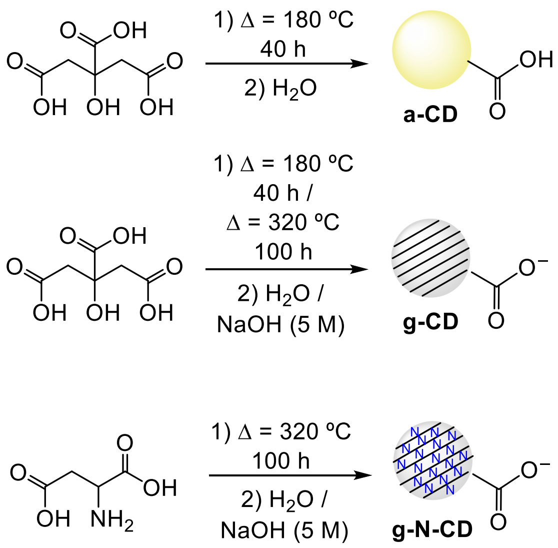

Scheme S1. Procedure for the synthesis of the carbon dots for this study.

2. Tables

Table S1. Summary of separated fractions and sizes of the GF-SEC separated CDs.

	GF-SEC fractions	TEM particle size (nm)
a-CD	S1	>7.6
	S2	7.6 ± 0.8
	S3	6.6 ± 0.3
	S4	4.6 ± 0.4
	S5	3.9 ± 0.3
	S6	3.2 ± 0.1
	S7	3.1-2.3 ± 0.3
	S8	2.2 ± 0.1
	S9	2.0 ± 0.1
	S10	<2.0
	S11	<2.0
g-CD	S1	>6.1
	S2	6.1 ± 0.3
	S3	4.7 ± 0.3
	S4	4.0 ± 0.4
	S5	3.1 ± 0.4
	S6	3.0 ± 0.1
	S7	2.9 ± 0.1
	S8	2.7 ± 0.1
	S9	2 ± 0.1
	S10	<2.0
	S11	<2.0
g-N-CD	S1	>4.4
	S2	4.4 ± 0.2
	S3	3.8 ± 0.4
	S4	3.2 ± 0.4
	S5	3.1 ± 0.4
	S6	2.9 ± 0.4
	S7	2.1 ± 0.2
	S8	2.0 ± 0.1
	S9	<2.0
	S10	<2.0
	S11	<2.0

Average size for CDs as synthesized are: i) 6.4 ± 2.1 nm for a-CD, ii) 3.2 ± 1.1 nm for g-CD and 3.0 ± 1.1 nm for g-N-CD. A HiLoad® 26/600 Superdex® 200 pg prepacked column (Cytiva) was used to purify the CDs in 20 mM Borate buffer (pH 8), and the column was eluted at a flow rate of 2 mL min^{-1}.

Table S2. DLS and zeta potential measurements of the eluted size separated CDs.

	TEM particle size (nm)	DLS measured size (nm)[a]	Zeta potential (mV)
a-CD	7.6 ± 0.8	130.6 ± 43.3	–19.4 ± 1.5
	6.6 ± 0.3	109.5 ± 19.0	–25.9 ± 1.6
	4.6 ± 0.4	89.9 ± 20.2	–23.2 ± 2.1
	2.2 ± 0.3	87.0 ± 33.1	–42.5 ± 2.4
g-CD	6.1 ± 0.3	465.6 ± 12.7	–38.5 ± 1.4
	4.7 ± 0.3	332.8 ± 14.1	–39.1 ± 1.4
	3.1 ± 0.4	10.9 ± 7.9	–50.0 ± 1.4
	2.7 ± 0.3	6.4 ± 3.1	–53.7 ± 1.3
	2 ± 0.1	2.8 ± 0.6	–52.7 ± 4.3
g-N-CD	4.4 ± 0.2	198.4 ± 83.6	–21.0 ± 1.3
	3.8 ± 0.4	116.3 ± 17.7	–30.7 ± 1.6
	3.2 ± 0.4	65.1 ± 28.1	–37.2 ± 0.9
	2.9 ± 0.4	56.3 ± 27.4	–40.2 ± 1.5

Average zeta potential for CDs as synthesized are: i) –17.0 ± 1.0 mV for a-CD, ii) –26.8 ± 1.4 mV for g-CD and –23.0 ± 1.0 mV for g-N-CD. A HiLoad® 26/600 Superdex® 200 pg prepacked column (Cytiva) was used to purify the CDs in 20 mM Borate buffer (pH 8), and the column was eluted at a flow rate of 2 mL min^{-1}. [a] It is worth mentioning that CD solutions, in 20 mM Borate buffer (pH 8), tend to aggregate in the DLS cuvette while measuring since there is no stirring, so the measures should be taken as an idea of the general trend observed for the particle size decrease, not the real particle diameter.

Table S3. Singlet vertical electronic excitation energy, oscillator strengths (*f*), CI coefficients and primary character (|*CI*| > 0.2) based on the frontier molecular orbitals calculated for the 4×4-1L system. Only transitions with finite oscillator strengths are shown.

Transition	Energy	*f*	Composition	CI coefficients
$S_0 \rightarrow S_1$	1.64 eV<br>754.06 nm	0.7459	HOMO – LUMO	0.69925
$S_0 \rightarrow S_4$	3.04 eV<br>407.38 nm	0.0008	HOMO – LUMO+3<br>HOMO-3 – LUMO	0.50306<br>0.45064

Table S4. Singlet vertical electronic excitation energy, oscillator strengths (*f*), CI coefficients and primary character (|*CI*| > 0.2) based on the frontier molecular orbitals calculated for the 4×4-4L system. Only transitions with finite oscillator strengths are shown.

Transition	Energy	*f*	Composition	CI coefficients
$S_0 \rightarrow S_2$	1.45 eV	0.0171	HOMO – LUMO+1	0.47753
	857.25 nm		HOMO-2 – LUMO	0.29614
			HOMO – LUMO	0.26071
			HOMO-1 – LUMO+1	0.23007
$S_0 \rightarrow S_4$	1.53 eV	0.2662	HOMO – LUMO	0.49871
	812.68 nm		HOMO-1 – LUMO+1	0.33040
			HOMO – LUMO+2	0.23302
			HOMO-3 – LUMO+1	0.21407
$S_0 \rightarrow S_5$	1.60 eV	0.3021	HOMO-3 – LUMO+1	0.45178
	775.21 nm		HOMO-2 – LUMO	0.29778
			HOMO – LUMO	0.29049
			HOMO – LUMO+2	0.24075
$S_0 \rightarrow S_8$	1.70 eV	0.0308	HOMO-1 – LUMO+3	0.47974
	728.45 nm		HOMO-2 – LUMO+2	0.31294
			HOMO-2 – LUMO	0.30935
			HOMO-1 – LUMO+1	0.22340
$S_0 \rightarrow S_{10}$	1.90 eV	1.0584	HOMO-3 – LUMO+3	0.47556
	653.85 nm		HOMO-2 – LUMO+2	0.40227
			HOMO-1 – LUMO+1	0.26220
$S_0 \rightarrow S_{12}$	2.24 eV	0.0075	HOMO+1 – LUMO+2	0.33207
	552.85 nm		HOMO – LUMO+2	0.32994
			HOMO-3 – LUMO+1	0.26060
			HOMO-1 – LUMO+3	0.26044
			HOMO – LUMO	0.25213
			HOMO-3 – LUMO+3	0.25401

3. Figures

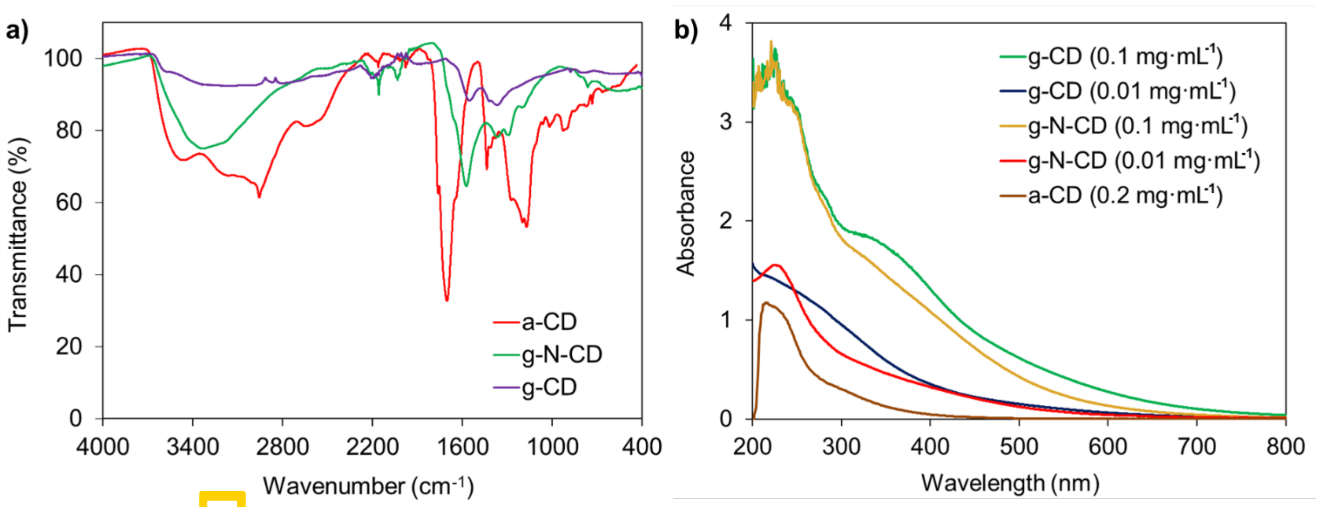

Figure S1. (**a**) IR and [illegible]UV-vis characterization of the synthesized a-CD, g-CD and g-N-CD in H_2O at 298 K.

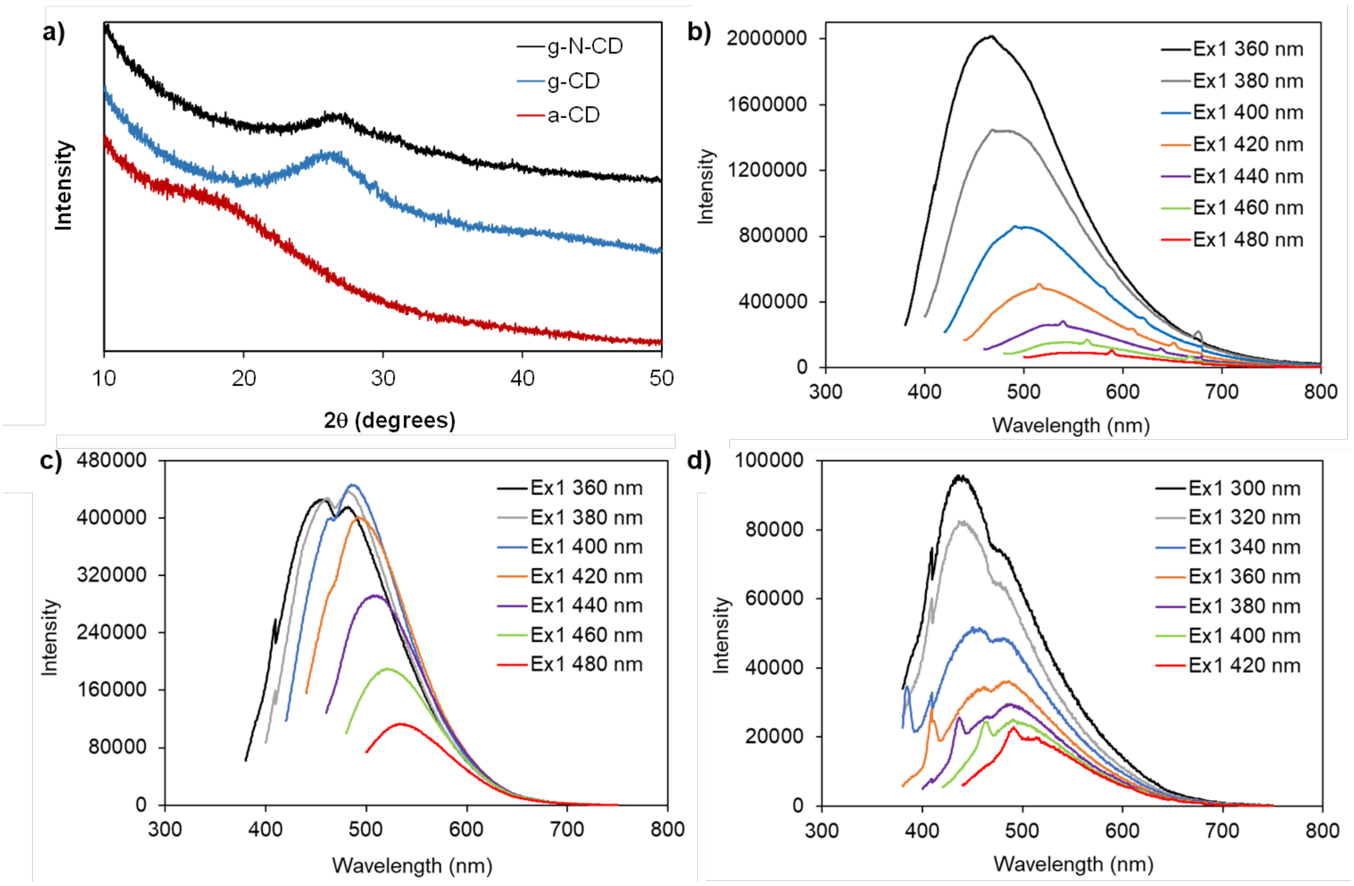

Figure S2. Powder X-Ray diffraction (XRD) ~~patters~~ of g-N-CD (black line), g-CD (blue line) and a-CD (red line) (**a**), and emission spectra of the synthesized (**b**) a-CD, (**c**) g-CD and (**d**) g-N-CD in H_2O at 298 K at different excitation wavelengths (see legends). Note: The powder XRD pattern for g-N-CD and g-CD show a broad but well-defined peak consistent with a nanocrystalline graphitic structure (graphitic core). For g-N-CD the peak is centered at *ca.* 27.0° 2θ, which corresponds to the (002) reflection in graphitic structures and a lattice spacing of 3.25 Å (d_{002} = 3.35 Å in bulk graphite).[1-2] For g-CD there is a well-defined peak (200) centered at 26.8° 2θ (lattice spacing of 3.29 Å) confirming the nanocrystalline graphitic core in g-CD. a-CD do not display this feature, in agreement with their predominant amorphous carbon core.[3]

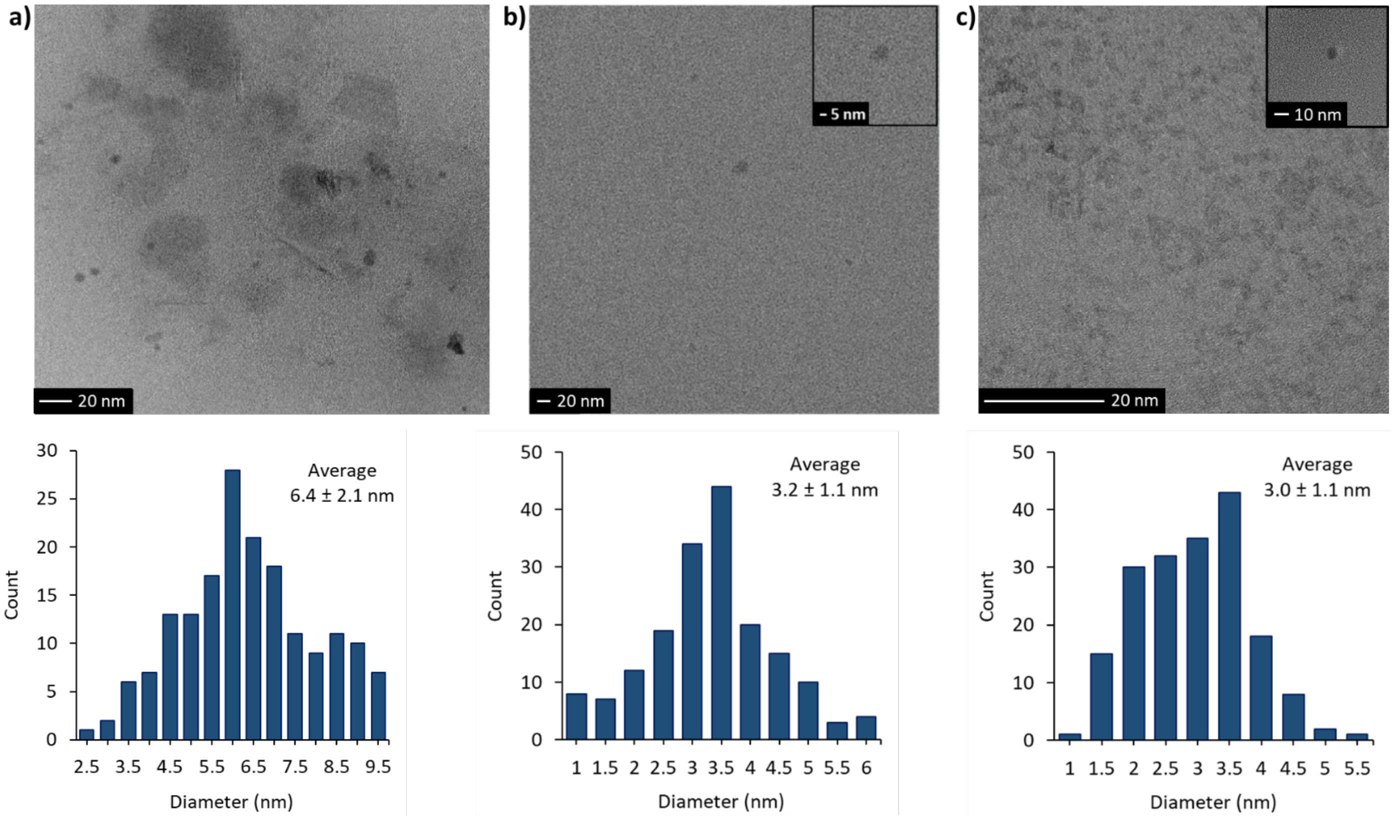

Figure S3. TEM images and ~~the~~ distribution of particle sizes of the synthesized (**a**) a-CD, (**b**) g-CD and (**c**) g-N-CD bulk materials before purification.

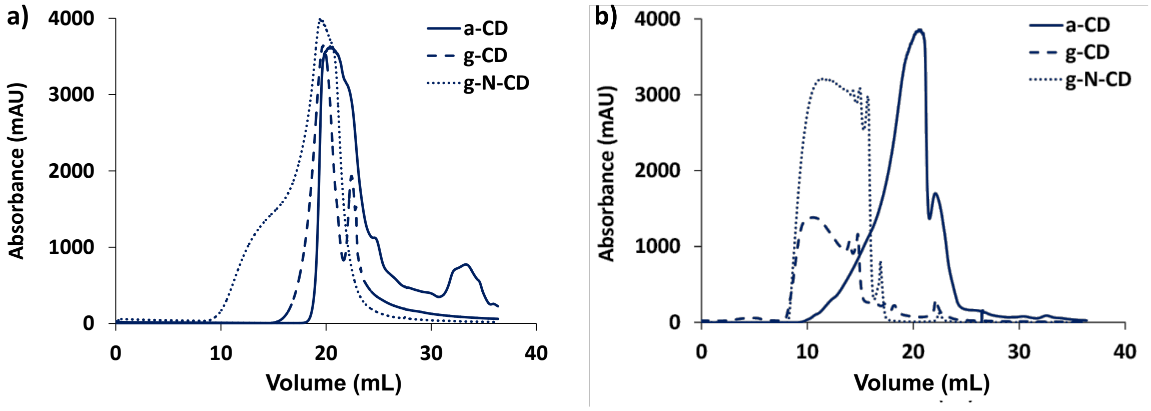

Figure S4. GF-SEC traces of the separation of a-CD, g-CD and g-N-CD in (**a**) tris·HCl buffer pH 7.3 (100 mM) NaCl (100 mM) and (**b**) borate buffer pH 8 (20 mM) using a Superdex 200 pg Increase 10/300 GL SEC column. Broad GF traces with tris·HCl buffer pH 7.3 (100 mM) NaCl (100 mM) suggest interaction between the column matrix and the CDs. Note: According to SEC principles, a-CDs should elute earlier than g-CD and g-N-CD due to their bigger size. The elution times can therefore be explained by the stronger interactions between the a-CDs and the dextran gel matrix compared to the other CDs, even when using borate buffer as eluent. Importantly, these CD-gel interactions do not change for the same type of CDs with different sizes and we can still employ GF-SEC for size-separation. This is supported by our TEM analyses and confirms separation between 2 and 7.6 nm, within the calculated pore size of the resin.

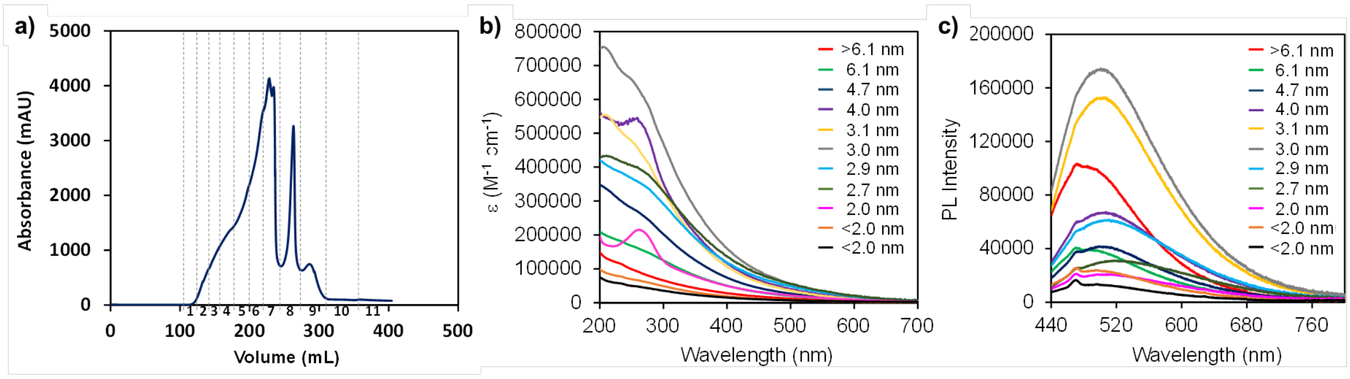

Figure S5. (**a**) Scale-up GF-SEC traces of the separation of g-CD in borate buffer pH 8 (20 mM) using a Superdex 200 pg Hi Load 26/600 GL. (**b**) UV-vis and (**c**) emission spectra (λ_{ex} = 405 nm) of the GF SEC separated fractions of g-CD (ranging from > 6.1 to < 2 nm size as described in the legend) in borate buffer pH 8 (20 mM) at 25 ºC.

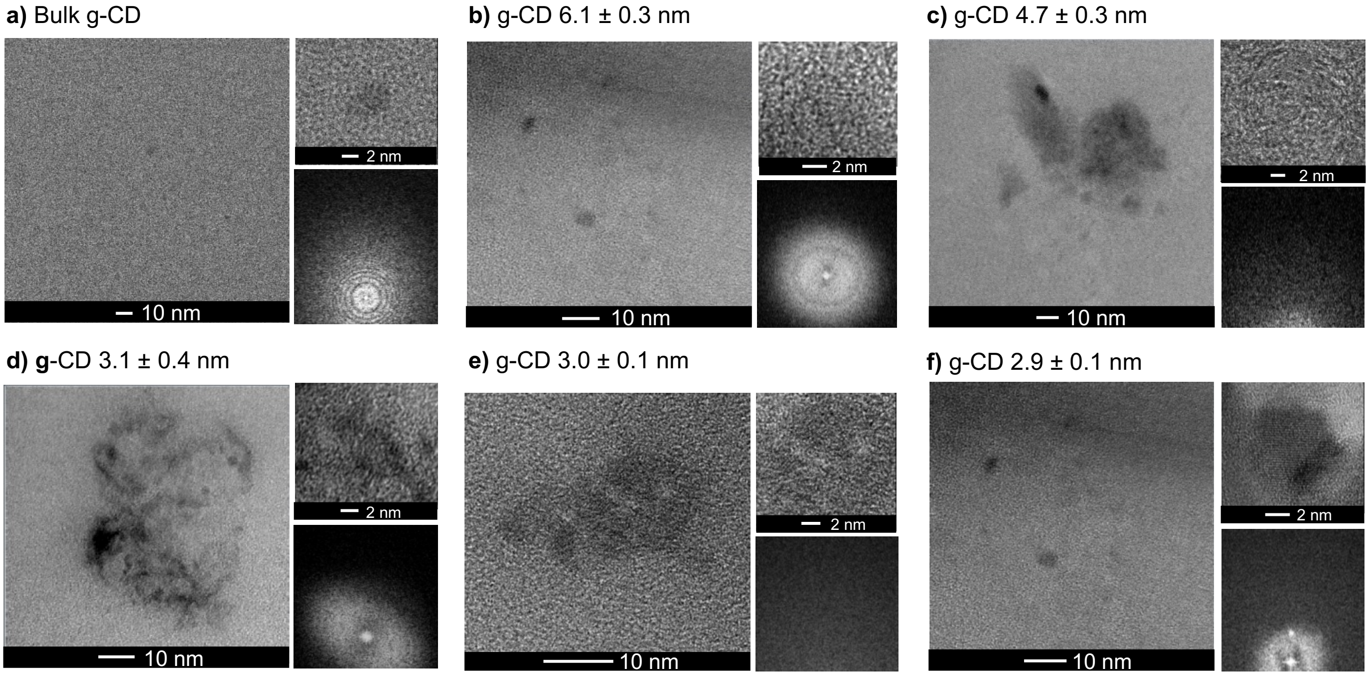

Figure S6. TEM images and corresponding FFT pattern of the bulk g-CD (**a**) and the different size-separated fractions (**b-f**).

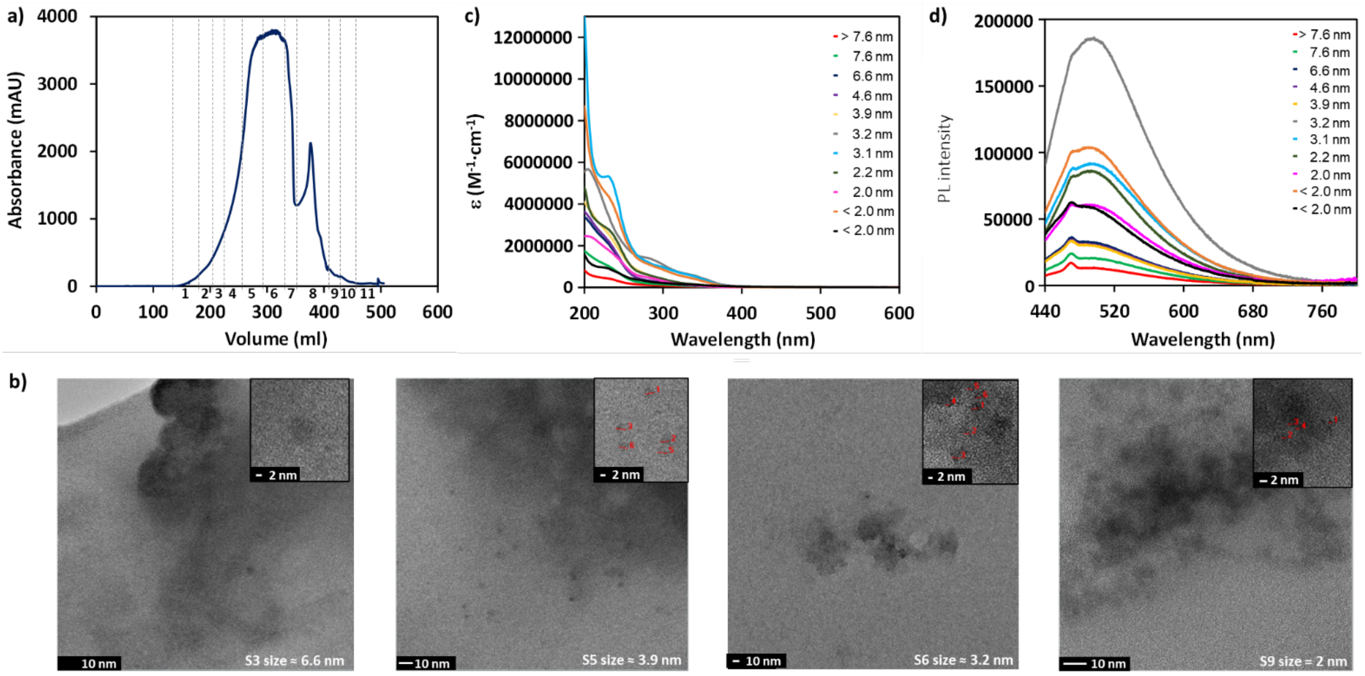

Figure S7. Scale-up GF-SEC traces of the separation of a-CD in borate buffer pH 8 (20 mM) using a Superdex 200 pg Hi Load 26/600 GL (**a**), TEM images (**b**), UV-vis spectra (**c**), and emission spectra (λ_{ex} = 405 nm) (**d**) of the separated fractions (0.14 mg·mL^{-1} concentration). Color codes correspond to the color band associated to each fraction. Red lines in the TEM images show an individual particle.

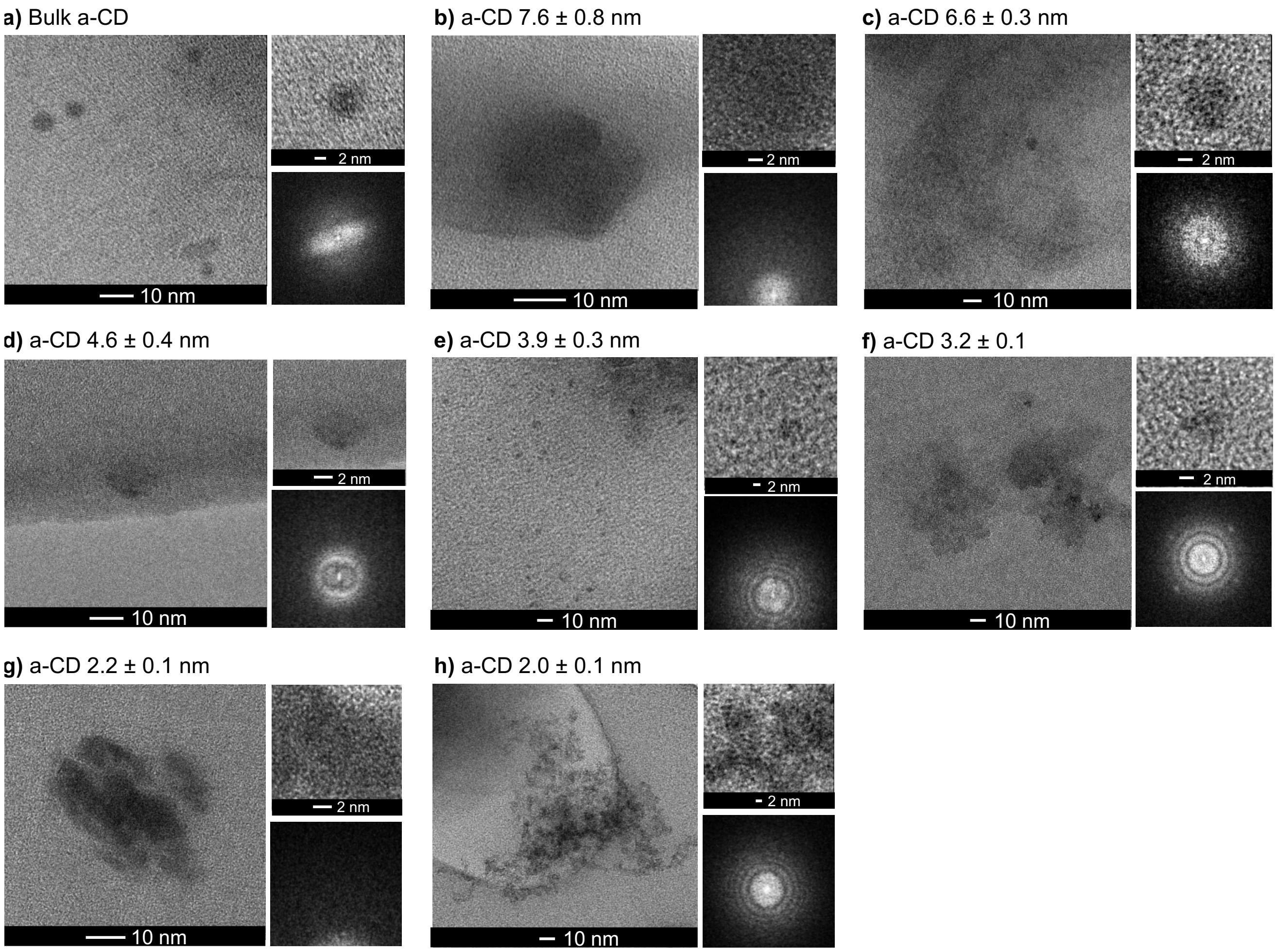

Figure S8. TEM images and corresponding FFT pattern of the bulk a-CD (**a**) and the different size-separated fractions (**b-h**).

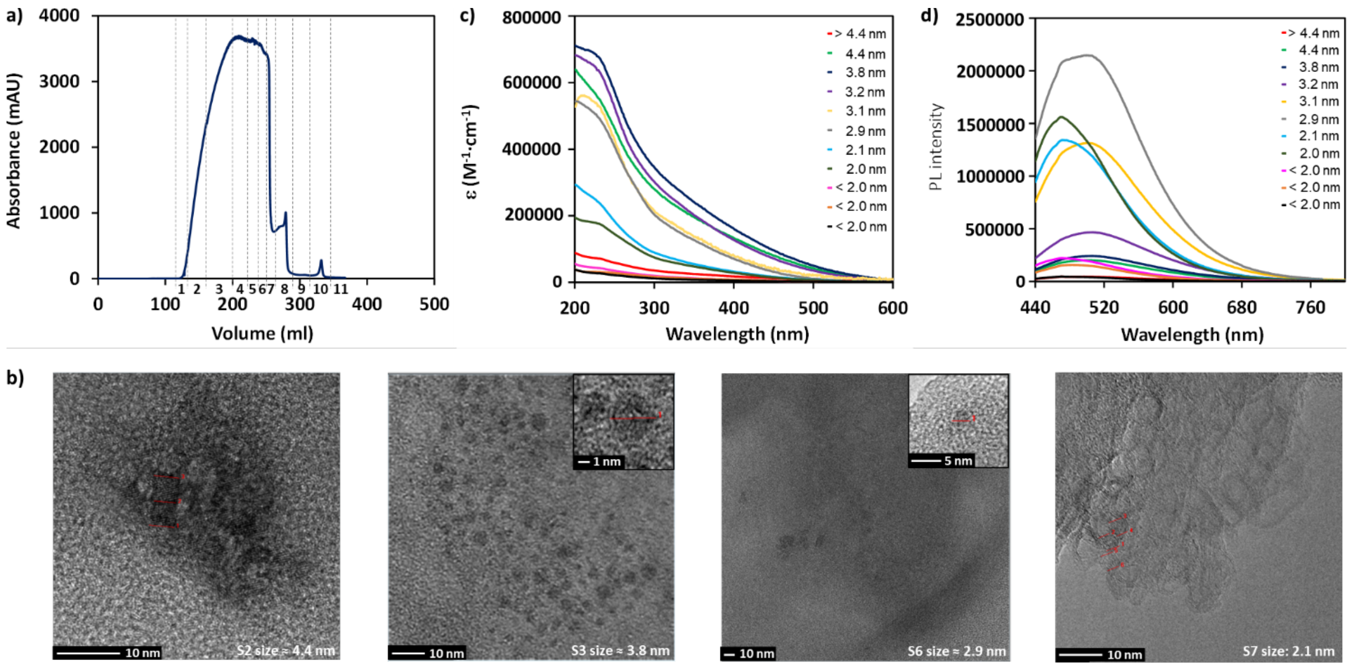

Figure S9. Scale-up GF-SEC traces of the separation of g-N-CD in borate buffer pH 8 (20 mM) using a Superdex 200 pg Hi Load 26/600 GL (**a**), TEM images (**b**), UV-vis spectra (**c**), and emission spectra (λ_{ex} = 405 nm) (**d**) of the separated fractions (0.14 mg·mL^{-1} concentration). Color codes correspond to the color band associated to each fraction.

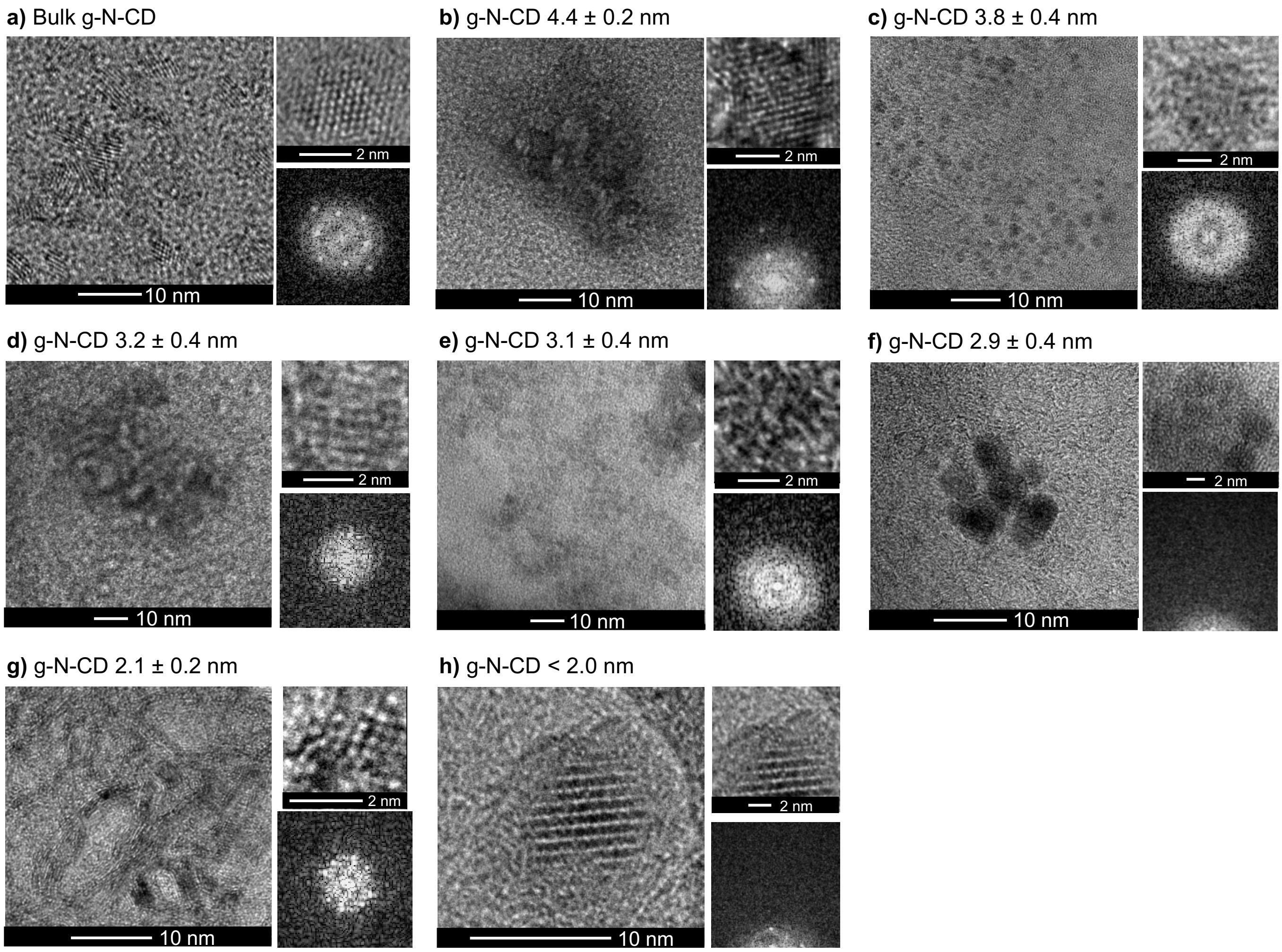

Figure S10. TEM images and FFT pattern of the bulk g-N-CD (**a**) and the different size-separated fractions (**b-h**).

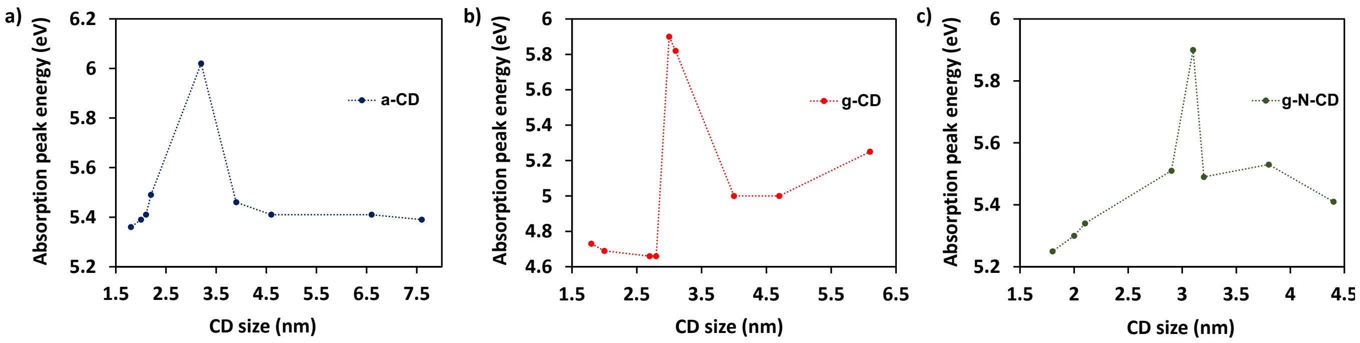

Figure S11. Absorption peak energy as function of the CDs size for (**a**), a-CD (**b**), g-CD and (**c**) g-N-CD.

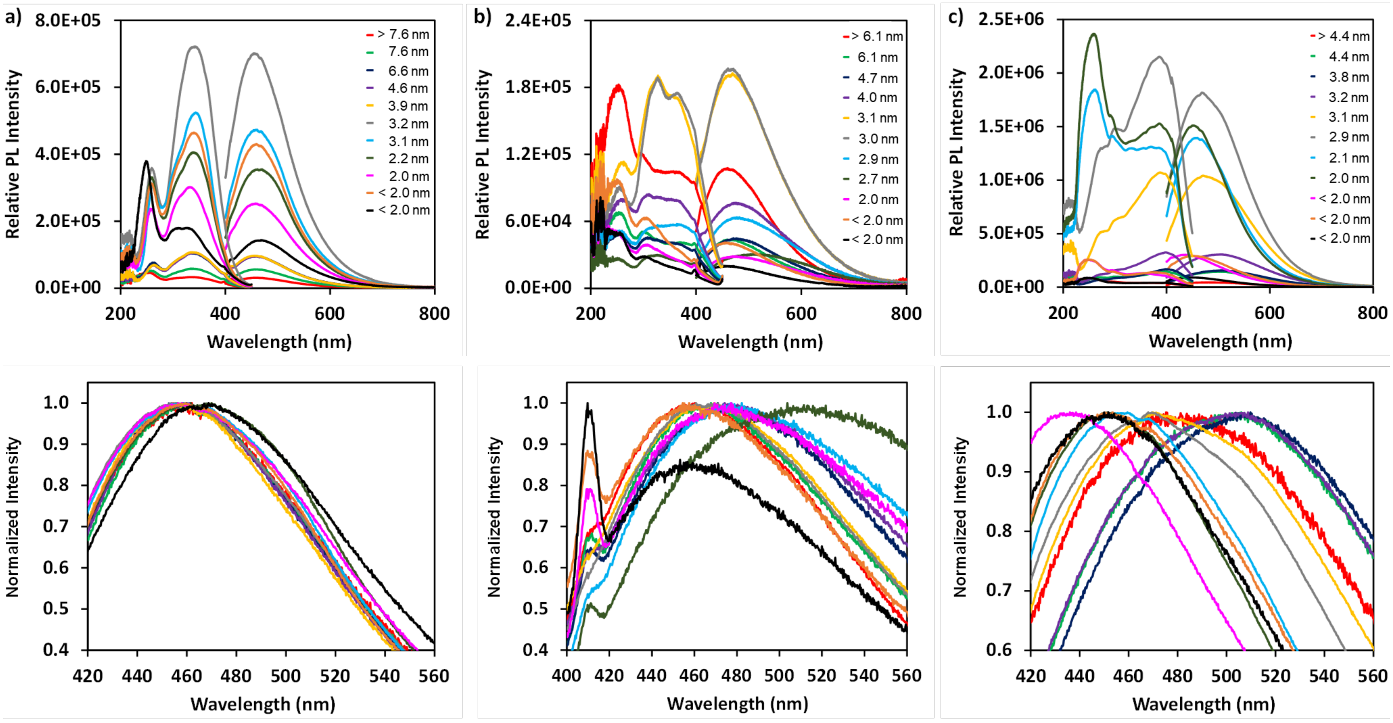

Figure S12. Excitation (λ_{em} = 460 nm) and emission spectra (λ_{ex} = 360 nm) with a magnified region of the emission spectra of the separated fractions (**a**) a-CD, (**b**) g-CD and (**c**) g-N-CD.

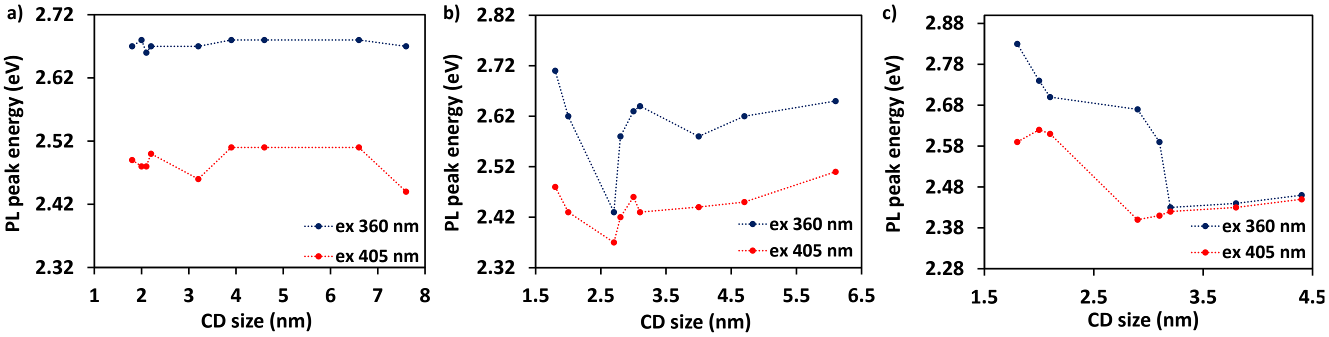

Figure S13. Photoluminescent (PL) peak energy shift as function of the CDs size at 360 and 405 nm excitation for (**a**), a-CD (**b**), g-CD and (**c**) g-N-CD.

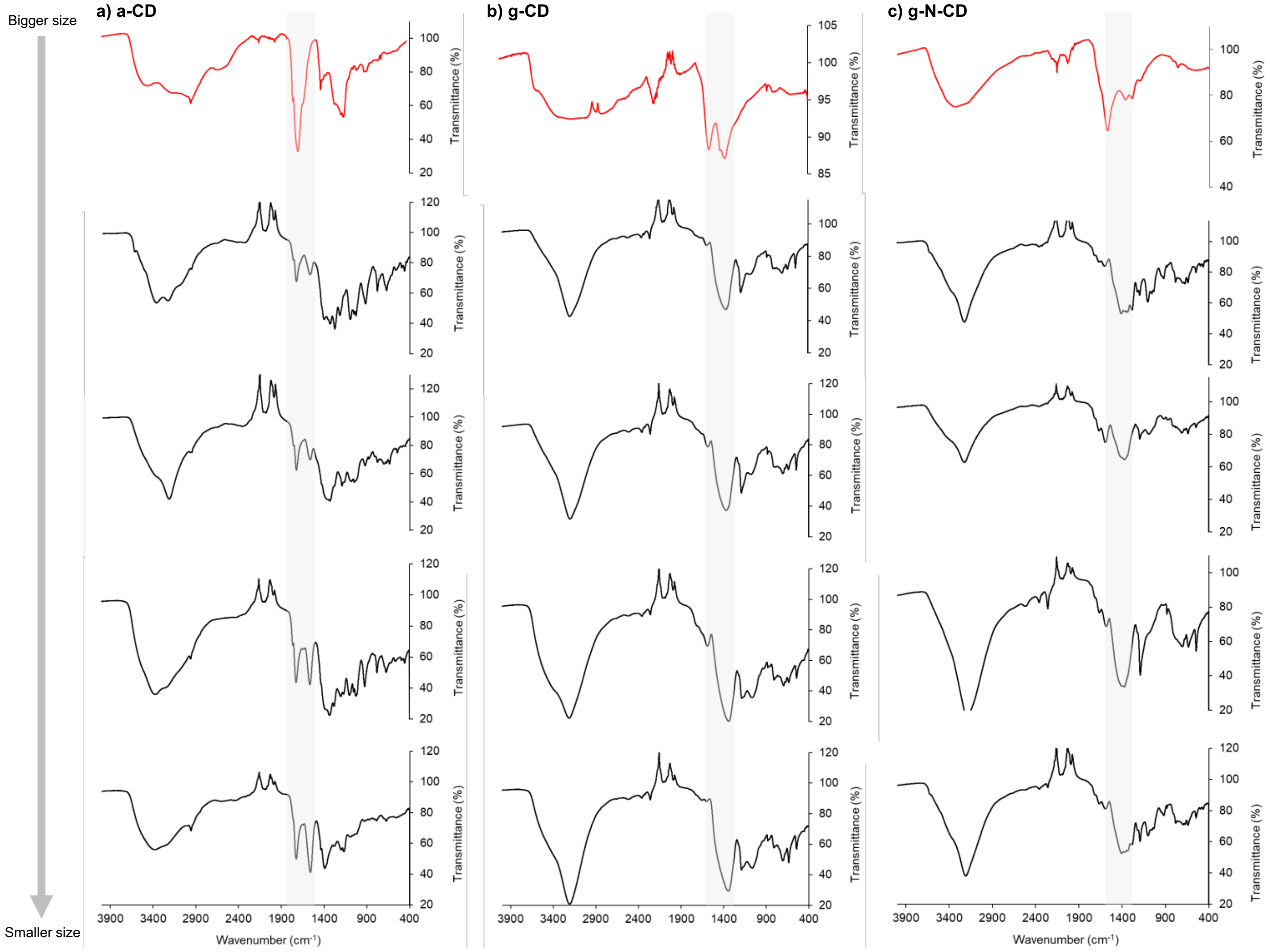

Figure S14. FTIR spectra of the bulk CDs (*top* spectra of **a**, **b** and **c**) and of some of the size separated fractions (from bigger to smaller) for (**a**), a-CD (**b**), g-CD and (**c**) g-N-CD.

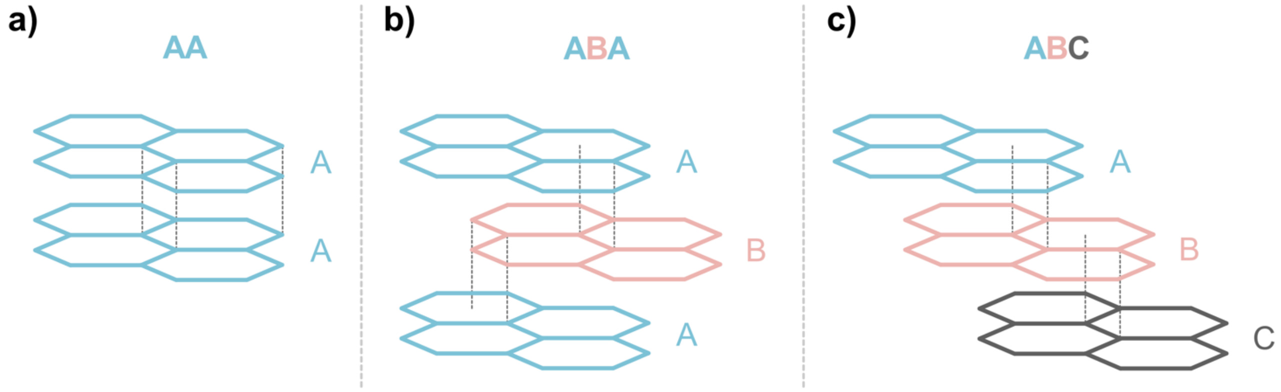

Figure S15. Schematic representation of the (a) AA, (b) ABA, and (c) ABC packings in a 2×2 graphene model system.

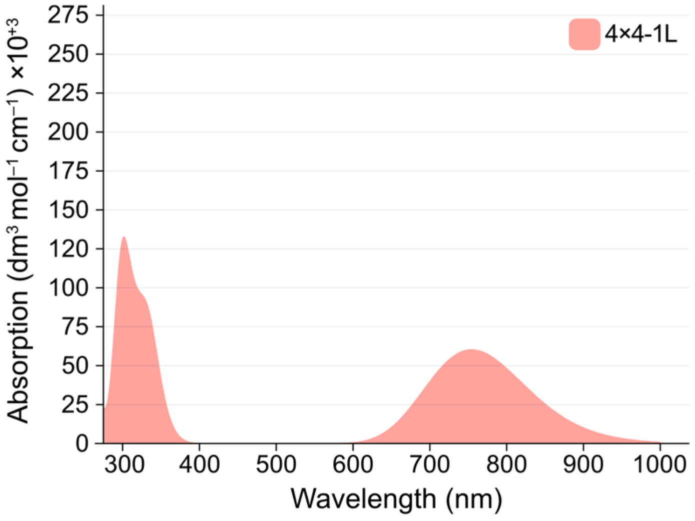

Figure S16. Calculated optical adsorption spectra for the 4×4-1L system.

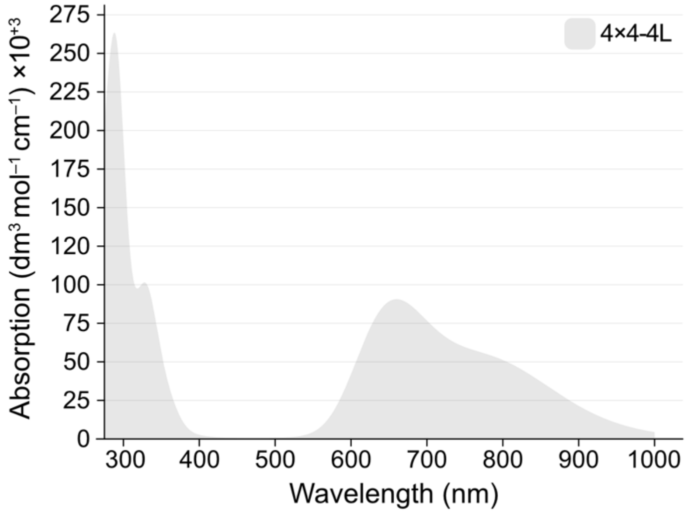

Figure S17. Calculated optical adsorption spectra for the 4×4-4L system.

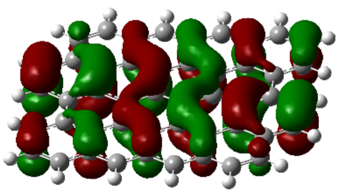<br>HOMO-3<br>-7.93 eV	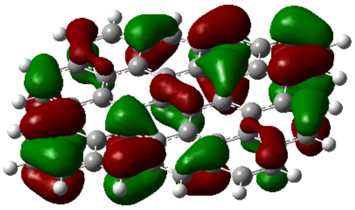<br>HOMO-2<br>-7.78 eV	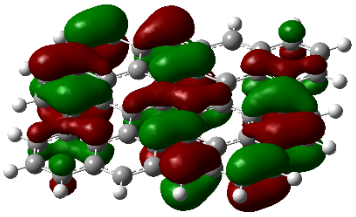<br>HOMO-1<br>-7.25 eV
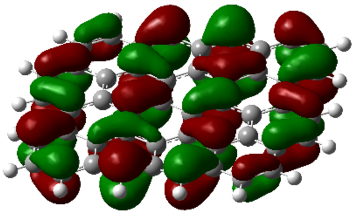<br>HOMO<br>-5.65 eV	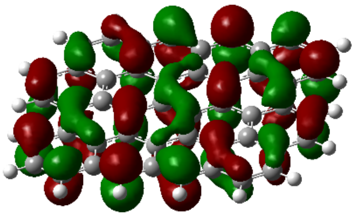<br>LUMO<br>-1.70 eV	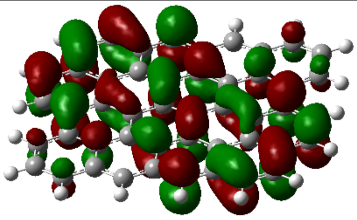<br>LUMO+1<br>-0.004 eV
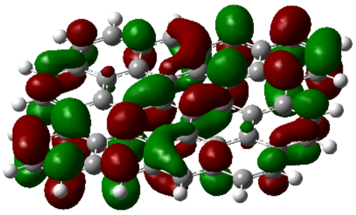<br>LUMO+2<br>0.41 eV	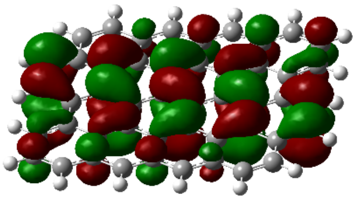<br>LUMO+3<br>0.54 eV	

Figure S18. Frontier molecular orbitals involved in the TD-DFT vertical excitations of 4×4-1L.

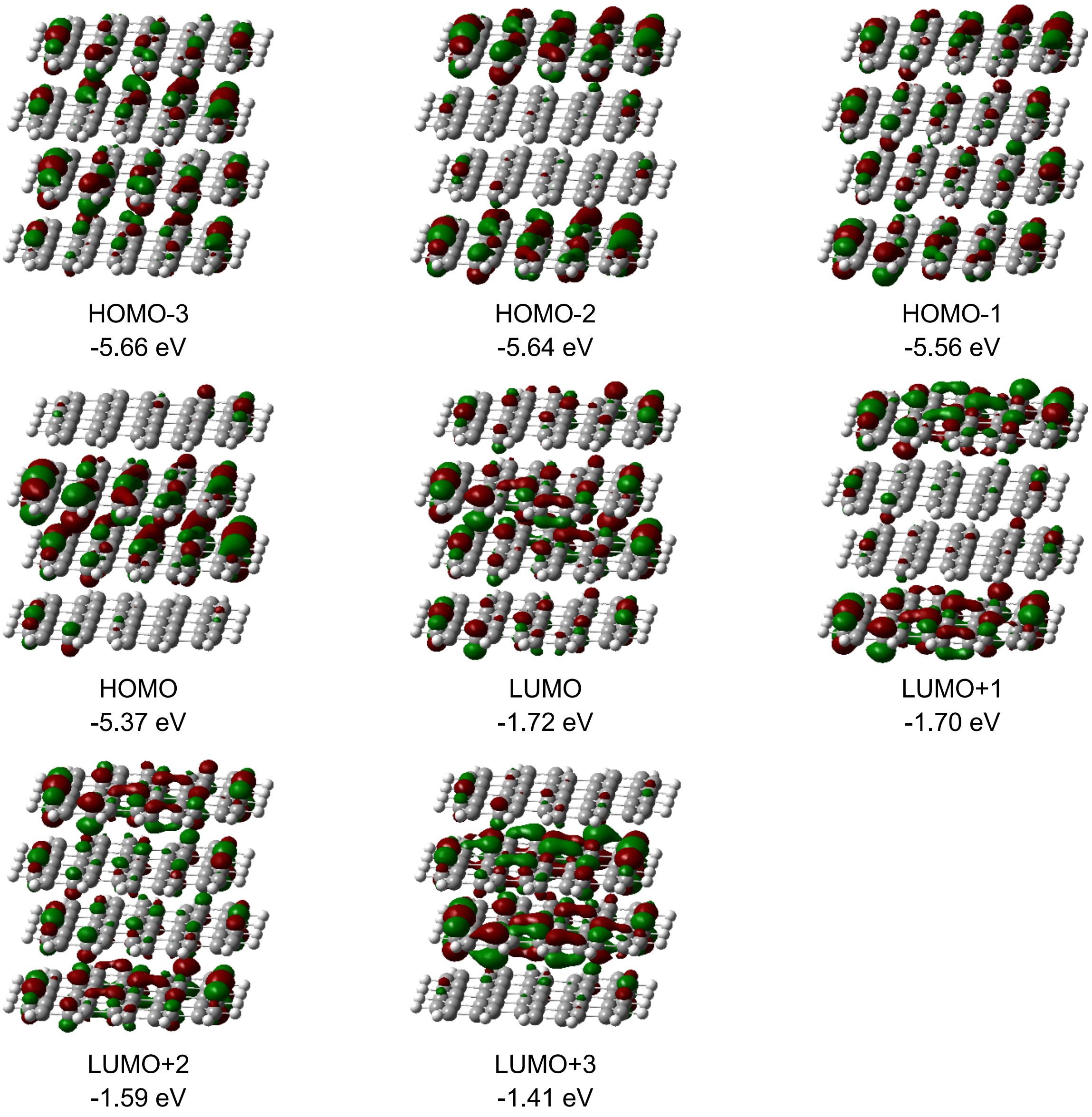

Figure S19. Frontier molecular orbitals involved in the TD-DFT vertical excitations of 4×4-4L.

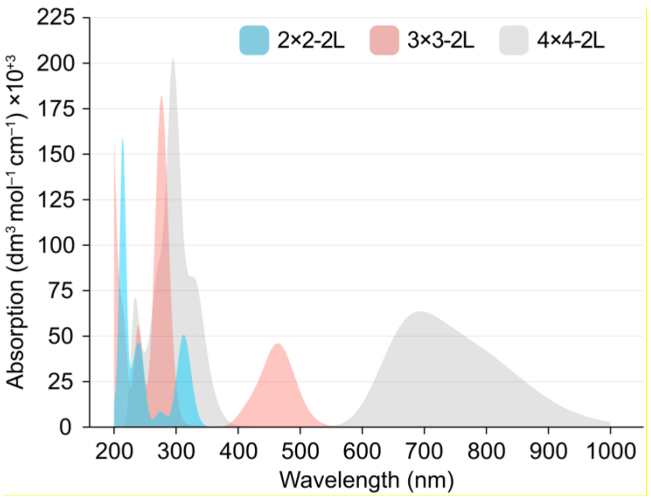

Figure S20. Simulated absorption spectra of graphene systems with varying sizes and two atomic layers.

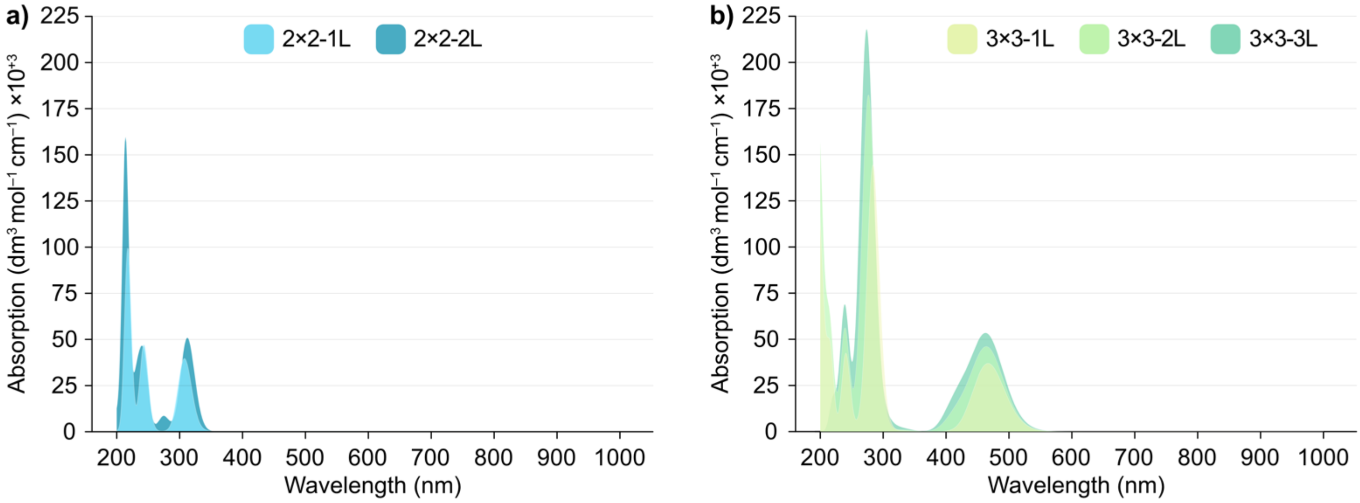

Figure S21. Simulated absorption spectra of the systems containing 2×2 (***a***) and 3×3 (***b***) graphene sheets with different number of layers.

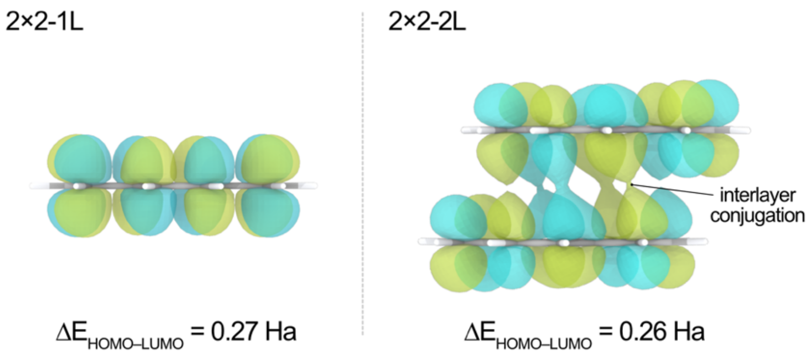

Figure S22. Side view representation of the isosurfaces (isovalue = 0.02 a.u.) of the LUMO of the 2×2 system with different number of layers. The calculated HOMO-LUMO gap ($\Delta E_{HOMO\text{-}LUMO}$) is also provided.

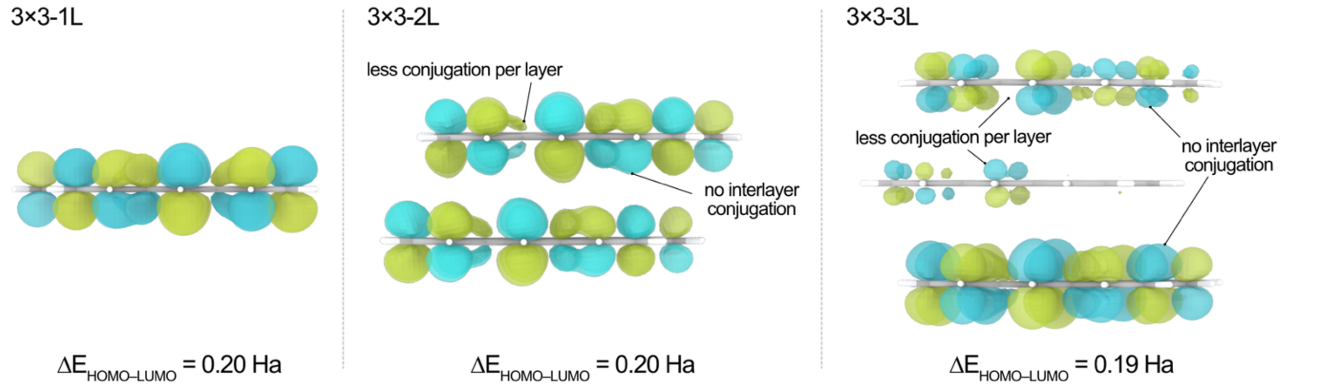

Figure S23. Side view representation of the isosurfaces (isovalue = 0.02 a.u.) of the LUMO of the 3×3 system with different number of layers. The calculated HOMO-LUMO gap ($\Delta E_{HOMO-LUMO}$) is also provided.

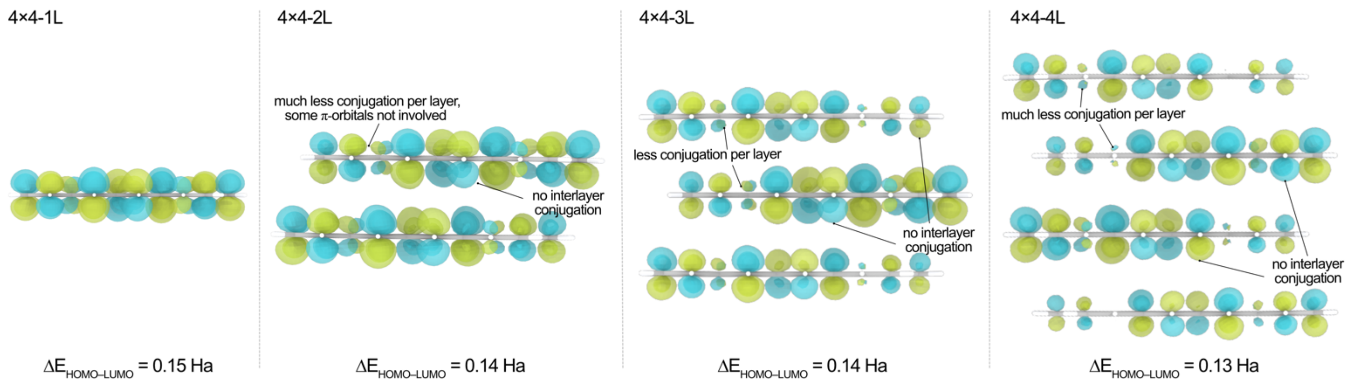

Figure S24. Side view representation of the isosurfaces (isovalue = 0.02 a.u.) of the LUMO of the 4×4 system with different number of layers. The calculated HOMO-LUMO gap ($\Delta E_{HOMO-LUMO}$) is also provided.

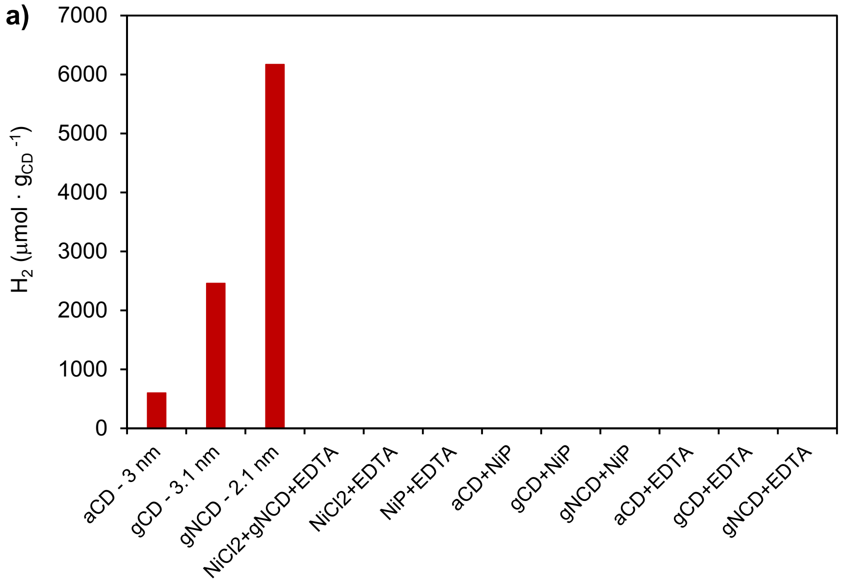

b)

Sample	[EDTA] (M)	H_2 (μmol g_{CD}^{-1})
g-CD 2 nm	0.1	102.5 ± 84.1
	0.2	109.25 ± 0.7
g-N-CD 3.8 nm	0.05	2492.8 ± 16.8
	0.1	2321.5 ± 0.0
g-N-CD 3.1 nm	0.05	2368.7 ± 17.1
	0.1	2597.7 ± 173.6
g-N-CD 2 nm	0.1	500.6 ± 148.8
	0.2	364.0 ± 4.9

Figure S25. (**a**) H_2 evolution studies with the optimum size for each type of CD and control studies without **NiP**, using **NiP** without CDs, and using $NiCl_2$ as control. (**b**) Experiments with different concentration of EDTA for the selected best and worst performing CDs. Catalytic conditions: a-CD, g-CD and g-N-CD (0.5 mg), **NiP** (50 nmol), $NiCl_2$ (30 nmol) and EDTA (0.1 M, pH 6, unless otherwise indicated) in water irradiation at 405 ± 10 nm for 24 h, at 25 °C under N_2 atmosphere.

4. Supporting References

1. Peng, H.; Travas-Sejdic, J., Simple Aqueous Solution Route to Luminescent Carbogenic Dots from Carbohydrates. *Chem. Mater.* 2009, **21** (23), 5563-5565.
2. Zhou, J.; Booker, C.; Li, R.; Zhou, X.; Sham, T.-K.; Sun, X.; Ding, Z., An Electrochemical Avenue to Blue Luminescent Nanocrystals from Multiwalled Carbon Nanotubes (MWCNTs). *J. Am. Chem. Soc.* 2007, **129** (4), 744-745.
3. Martindale, B. C. M.; Hutton, G. A. M.; Caputo, C. A.; Prantl, S.; Godin, R.; Durrant, J. R.; Reisner, E., Enhancing Light Absorption and Charge Transfer Efficiency in Carbon Dots through Graphitization and Core Nitrogen Doping. *Angew. Chem. Int. Ed.* 2017, **56** (23), 6459-6463.

End of Supporting Information
